# Sampling-Based Real-Time Motion Planning under State Uncertainty for Autonomous Micro-Aerial Vehicles in GPS-Denied Environments

**DOI:** 10.3390/s141121791

**Published:** 2014-11-18

**Authors:** Dachuan Li, Qing Li, Nong Cheng, Jingyan Song

**Affiliations:** 1 Department of Automation, Tsinghua University, Bejing 100084, China; E-Mails: liqing@tsinghua.edu.cn (Q.L.); ncheng@tsinghua.edu.cn (N.C.); jysong@tsinghua.edu.cn (J.S.); 2 National Key Laboratory on Flight Vehicle Control Integrated Technology, Flight Automatic Control Research Institute, Xi'an 710065, China

**Keywords:** motion planning, micro-aerial vehicles, rapidly exploring random trees (RRT), state estimation uncertainty

## Abstract

This paper presents a real-time motion planning approach for autonomous vehicles with complex dynamics and state uncertainty. The approach is motivated by the motion planning problem for autonomous vehicles navigating in GPS-denied dynamic environments, which involves non-linear and/or non-holonomic vehicle dynamics, incomplete state estimates, and constraints imposed by uncertain and cluttered environments. To address the above motion planning problem, we propose an extension of the closed-loop rapid belief trees, the closed-loop random belief trees (CL-RBT), which incorporates predictions of the position estimation uncertainty, using a factored form of the covariance provided by the Kalman filter-based estimator. The proposed motion planner operates by incrementally constructing a tree of dynamically feasible trajectories using the closed-loop prediction, while selecting candidate paths with low uncertainty using efficient covariance update and propagation. The algorithm can operate in real-time, continuously providing the controller with feasible paths for execution, enabling the vehicle to account for dynamic and uncertain environments. Simulation results demonstrate that the proposed approach can generate feasible trajectories that reduce the state estimation uncertainty, while handling complex vehicle dynamics and environment constraints.

## 1. Introduction

Autonomous micro-aerial vehicles (MAV) are playing an increasingly important role in many civil and military applications. MAVs that are capable of autonomously making decisions and operating can be applied in many tasks scenarios that are not accessible by humans and ground mobile robots, such as indoor exploration and mapping, search and rescue, disaster relief, *etc.* As a result, there has been an increasing interest in developing autonomous MAV systems for indoor navigation. In recent years, many researchers have developed and implemented various kinds of MAV systems demonstrating various degrees of autonomy in performing tasks, such as indoor exploration [1], environment mapping [2] and agile flight [3].

Despite the considerable progress achieved in this domain, there are still many challenges in developing fully autonomous MAV systems. One of the key problems is the motion/path planning for MAVs capable of operating in GPS-denied complex environments. For MAVs performing tasks in such scenarios, the motion planning algorithm must comply with constraints, including complex vehicle dynamics (non-linear and/or non-holonomic dynamics with a high dimensional state space) and environmental constraints (unstructured and uncertain, time-varying operating environments). Particularly, since MAVs typically cannot directly get access to the information of the current state, they must estimate the distribution over the states using measurements from onboard sensors or external aids (GPS [4], external motion capture system [5]). However, GPS is unreliable in urban canyons and completely unavailable in most indoor environments; the external motion capture system is also impractical for such scenarios, since it requires pre-installation of camera arrays in the environment. As a result, the state estimation of MAVs must rely only on onboard sensing capabilities, which is highly constrained in terms of precision and range due to the size and weight constraints of the MAVs. Moreover, the performance of MAV state estimation and localization using exteroceptive sensors (laser rangefinder [6], camera [7] and RGB-D sensor [8]) varies across the environment depending on the distinctive features and perceptual structure of the environments. As a result, the motion planning progress must also integrate the uncertainty of state estimation to ensure reliability and robustness to imperfect and noisy state estimates caused by limited sensing capability. These challenges require that the motion/path planner must be able to generate feasible paths that satisfy the constraints imposed by uncertain and unstructured environments, as well as complex vehicle dynamics, while ensuring measurement gathering along the path to reduce state estimation uncertainty.

In this paper, we propose a real-time motion planning strategy (closed-loop random belief trees, CL-RBT) for autonomous vehicles with complex dynamics and in the presence of state estimation uncertainty, while handling the constraints imposed by the uncertain and dynamic operating environments. The proposed motion planning strategy is built upon the randomized sampling-based motion framework, the real-time closed-loop rapidly exploring random trees (CL-RRT) [9], which operates by sampling in the input space of the controller and generates trajectories through forward closed-loop simulation using the vehicle dynamics model and path-tracking controller. This allows the motion planner to easily account for non-linear and non-holonomic vehicle dynamics and dynamic uncertain environments. We extend the general CL-RRT to handle the state uncertainty by incorporating the prediction of posterior state estimation uncertainty in the motion planning framework. The uncertainty of state estimation is characterized using a factored form of the covariance [10] provided by the Kalman filter class-based estimator. This factored form enables efficient covariance propagation in that it combines the update of covariance along a path from multi-step observations into a single linear step, and the posterior covariance of a certain path after adding new nodes can also be updated online. The overall motion planning operates by incrementally constructing a tree of dynamically feasible trajectories in real time, while continuously selecting and executing trajectories that are a tradeoff between minimizing path cost and reducing localization uncertainty. We validated the proposed algorithm in various illustrative scenarios of a simulated quadrotor MAV with non-linear dynamics and limited sensing in enclosed and unstructured GPS-denied environments. Simulation results demonstrate that the CL-RBT can efficiently generate dynamically feasible trajectories that ensure state estimation accuracy, while preserving the property of the CL-RRT framework. The CL-RBT can handle complex vehicle dynamics and state uncertainty, enabling the MAV to autonomously navigate in uncertain, unstructured and GPS-denied environments.

The paper is organized as follows: Section 2 reviews related work on motion planning under uncertainty. Section 3 presents the formulation of the motion planning problem. Section 4 provides the covariance propagation approach. A detailed description of the CL-RBT is presented in Section 5, followed by the simulation results and analysis in Section 6. Finally, the paper is concluded in Section 7, with a discussion on future work.

## 2. Related Work

Conventionally, motion planning approaches under state uncertainty are typically formulated as a partially observable Markov decision process (POMDP) problem [11], which provides the most general mathematical framework for solving the planning problem with partial observability. While POMDP has been applied to low-dimensional and small-scale problems [12], most POMDP-based approaches rely on discretizing the state space, making it computationally intractable for realistic applications. Recently, many approaches have been proposed to address the problem of scalability using approximation and iterative methods. Van den Berg *et al.* [13] proposed an iterative motion planning approach to solve the continuous POMDP problem, using a belief space variant of the iterative LQG (linear-quadratic Gaussian) method. Similarly, Bai *et al.* [14] also formulated the problem as a continuous POMDP and solved the problem with a Monte Carlo value iteration method. However, these approaches are still less effective at addressing problems with complex vehicle dynamics and motion planning with large-scale, high dimensional state space or configuration space.

In contrast, sampling-based motion planning strategies have been widely acknowledged as effective approaches for solving motion planning problems with high dimensional configuration space and complex vehicle dynamics. In particular, the PRM (probabilistic roadmap) [15] and RRT (rapidly exploring random trees) [16] and their variants are the most widely applied sampling-based approaches, and they have been successfully applied in a number of applications. More recently, the closed-loop RRT [9,17,18] extends the conventional RRT by incorporating forward prediction using closed-loop dynamics model with controller. The CL-RRT can generate more feasible paths that account for complex vehicle dynamics, and it can be implemented in real-time to handle dynamic, uncertain environments. Several approaches have been proposed to incorporate state uncertainty in the sampling-based motion planning framework. Van den Berg *et al.* [19] proposed the LQG-MP strategy by integrating the *a priori* distribution over state estimates into the standard RRT framework. However, the LQG-MP algorithm is intractable for real-time applications, due to its high computation cost. In [20], Bry *et al.*, combined an LQG-based covariance pruning technique with the RRT* framework for planning in belief space. Alternative to RRT-based approaches, the belief roadmap (BRM) extends the PRM by integrating the predictions of state estimation uncertainty using an EKF (extended Kalman filter) estimator and performs belief planning by searching paths with the lowest uncertainty from the roadmap. The BRM was later extended to use the UKF estimator and heuristic sampling strategy [21], and it was further implemented on a quadrotor and proven successful for indoor flight tests [22]. However, the BRM assumes that the vehicle is fully controllable and treats the problem as a simple kinematic motion planning problem, e.g., it does not consider complex vehicle dynamics and the feasibility of the generated paths. As a result, it is not yet well suited to vehicles with complex dynamic constraints and dynamic environments. In addition, the BRM operates by planning on pre-constructed static graphs, and therefore, it is not a real-time planning algorithm in essence. Recent research has also considered another similar kind of planning problem with uncertainty, where the planner must generate paths that maximize information collection and reduce the uncertainty on the location estimates of features or targets in the environment. The information-rich RRT (IRRT) [23] addresses this path planning problem by incorporating the qualification of information gain into the CL-RRT framework, using the Fisher information matrix. This kind of problem can be regarded as the inverse problem of planning with uncertainty on the vehicle's own state.

## 3. Problem Formulation

### 3.1. Motion Planning under State Uncertainty

Consider a vehicle with non-linear dynamics that operates in an environment without external positioning systems. The vehicle typically does not have direct access to the accurate information of its current state, but instead obtains the estimates of the state using observations derived from the measurements of onboard sensors, which are typically noisy and incomplete. The stochastic dynamics and observation model of the system can be given by the following discrete time state-transition form:

(1) $$\begin{array}{ll} {\text{x}}_{t+1}=f({\text{x}}_{t},{\text{u}}_{t},{\text{w}}_{t}) & {\text{w}}_{t}\sim N(0,\text{Q})\\ {\text{z}}_{t}=f({\text{x}}_{t},{\text{v}}_{t}) & {\text{v}}_{t}\sim N(0,\text{R}) \end{array}$$

where **x**∈*X*,**u**∈*U* ∈ and **z**∈*Z* are the vehicle's state, control input and observation, respectively. **w***_t_* and **v***_t_* denote the process disturbance and measurement noise, and both can be formulated as Gaussian noise with zero mean and covariance (**Q** and **R**, respectively). Assuming that all probability distributions are Gaussian, given the previous observation (**z**_1:_*_t_*) and control inputs (**u**_1:_*_t_*), the state is typically estimated using a Bayesian filter, providing the distribution of the vehicle's state (or the belief of the state):

(2) $$p({\text{x}}_{t})=p({\text{x}}_{t}|{\text{u}}_{1:t},{\text{z}}_{1:t})$$

which is characterized by a mean state μ*_t_* and a covariance Σ*_t_*(*b*(**x***_t_*) = (*μ_t_*, Σ*_t_*)). The mean *μ_t_* provided by the Bayesian filter is a optimal estimation of the vehicle's state distribution, and it can be used for control and decision making, while the mean Σ*_t_* characterizes the confidence (or uncertainty) of state estimation.

Denoting *X_free_* as the subset of all collision-free states, given the initial state **x**_0_ ∈ *X_free_* and the goal region *X_goal_* ⊂ *X_free_* , as well as the partial knowledge of the environment, then the primary objective of motion planning is to find the control policy **u**_0:_*_T_* = π[*b*(**x**_0:_*_T_*)] and corresponding sequence of states **x**_0:_*_T_*, such that the vehicle reaches the goal region in a finite time horizon by applying the control policy:

(3) $${\text{x}}_{T}\in X_{\text{goal},}t\in \left(0,t_{f}\right],t_{f}\in (0,\infty )$$

meanwhile minimizing the following objective function:

(4) $$J(b({\text{x}}_{t}))=E_{b({\text{x}}_{\text{goal}})|b({\text{x}}_{t}),{\text{u}}_{0:T}}[C({\text{x}}_{t}|{\text{x}}_{\text{goal}})]+\sum_{t=0}^{T}C({\text{x}}_{t},{\text{u}}_{t})$$

where *C*(**x***_t_*|**x***_goal_*) is the expected cost function from **x***_t_* to **x***_goal_*; note that it takes the expectation form, since the measurement and state are both probabilistic, and *C* (**x***_t_*, **u***_t_*) is the cost by applying control **u***_t_*. The generated trajectory must also satisfy the constraints imposed by the vehicle dynamics Equation (1) and environments (*i.e.*, collision avoidance, **x**_0:T_∈*X_free_*). Moreover, when the vehicle does not have accurate knowledge of the estimate of its state, the motion planner must take the uncertainty of the state estimate along the generated paths into account, thus the motion planning problem into the belief planning problem (*i.e.*, planning in belief space [20]). By integrating the information from the mean and covariance of the belief, the motion planning algorithm must choose actions and beliefs, such that the posterior covariance at the end of the trajectory is minimized, yielding trajectories that achieve a trade-off between low path cost and high confidence in the state estimation.

### 3.2. State Estimation of Gaussian Systems

Consider a system with the dynamic and observation model in the form of Equation (1), one of the most common and robust methods for estimating the distribution over its state is the Bayesian filtering [10]. Given knowledge of the prior control input **u***_t_* and sensor measurement **z***_t+_*_1_, the belief of the state *b*(**x***_t_*_+1_) after a sequence of control and observation can be estimated as:

(5) $$b({\text{x}}_{t+1})=p({\text{x}}_{t+1}|{\text{u}}_{1:t},{\text{z}}_{1:t})=\lambda p({\text{z}}_{t+1}|{\text{x}}_{t+1})\int p({\text{x}}_{t+1}|{\text{x}}_{1:t},{\text{u}}_{1:t})b({\text{x}}_{t})d{\text{x}}_{t}$$

where λ is a normalization factor.

Assuming the state and observation transition function (*f*, *h*) are linear with state and observation, which are both Gaussian distributions; the Bayesian filtering can be implemented as the Kalman filter [24], while the extended Kalman filter (EKF) [25] is typically applied for estimating the state distribution of the system with nonlinear state and observation transition functions. For a system model of the form of Equation (1), the EKF state estimation can be divided into the process step and measurement update step. Denoting the distribution of the state *b*(**x***_t_*) = *N*(**x̂***_t_*, Σ*_t_*) , the process step first predicts the state using the control and state of the previous step, which is given as:

(6) $$\begin{array}{c} {\bar{\hat{\text{x}}}}_{t}=g({\hat{\text{x}}}_{t-1},{\text{u}}_{t})\\ {\bar{\text{∑}}}_{t}=G_{t}\sum_{t-1}G_{t}^{T}+V_{t}Q_{t}V_{t}^{T} \end{array}$$

where *G_t_* is the Jacobian of *g* with respect to the state, and *V_t_* is the Jacobian of *g* with respect to **w***_t_*. Then, the measurement step adjusts the estimate and covariance by incorporating the information from new observations:

(7) $$\begin{array}{c} {\hat{\text{x}}}_{t}={\bar{\hat{\text{x}}}}_{t}+K_{t}({\text{z}}_{t}-H_{t}{\bar{\hat{\text{x}}}}_{t})\\ {\text{∑}}_{t}={\bar{\text{∑}}}_{t}-K_{t}H_{t}{\bar{\text{∑}}}_{t} \end{array}$$

where *H_t_* denotes the Jacobian of *h* with respect to the state, and *K_t_* is the Kalman gain, which is updated by:

(8) $$K_{t}={\bar{\sum }}_{t}H_{t}^{T}{\left(R_{t}+H_{t}{\bar{\sum }}_{t}H_{t}^{T}\right)}^{-1}$$

Typically, the control and decision making are based on the mean **x̂***_t_* of the estimated distribution provided by the EKF, and the covariance, while the covariance Σ*_t_* captures the uncertainty of the state estimate using the sensor measurements. Therefore, the uncertainty of the state estimate along the trajectory from the path planner can be evaluated using the norm of the covariance, which will be discussed in the next section.

## 4. Linear Covariance Propagation

In order to evaluate the state uncertainty resulting from a specific planned trajectory, the most common approach for a system with the EKF estimator is to compute the posterior covariance of the ending belief *b*(**x***_t_*) from the sequence of actions and measurements along the trajectory. However, the propagation of the posterior covariance requires multiple iterative calculations of the EKF process and measurement updating from the initial belief according to Equation (4), leading to a heavy computational cost. This is even worse when the initial belief is modified, since the posterior covariance must be re-computed using the entire EKF updates from the new initial belief. In particular, this fact has a more significant effect on a sampling-based motion planner, since it generally constructs a graph and tree of multiple trajectories, thus different paths can result in different posterior covariance to the same node, this requires that the motion planner must perform the propagation process for each covariance.

To reduce the computational cost of the covariance propagation, we rely on previous results on the factorization of the covariance [10], which allows the propagation of posterior covariance to be propagated in a single linear update step instead of multiple non-linear updates for the EKF filter.

Following Theorem 1 in [10], the factorization of covariance is given by:

(9) $$\sum_{t}=\Lambda_{t}\prod_{t}^{-1}$$

where Λ*_t_* and П*_t_* can be calculated as linear functions of Λ*_t_*_−1_ and П*_t_*_−1_, using the EKF process and measurement update step.

The factorization of the covariance matrix can be proved using the following matrix inversion lemma:

Lemma 1. For matrices *M*_1_, *M*_2,_ *M*_3_∈ R*^n^*^×^*^n^* , we have:

(10) $$(M_{1}+M_{2}M_{3}^{-1})=M_{3}{\left(M_{2}+M_{1}M_{3}\right)}^{-1}$$

Denote the initial state covariance as Σ_0_, and Σ_0_ can be factored as:

(11) $$\sum_{0}=\sum_{0}{\text{I}}^{-1}$$

Given $\sum_{t-1}=\Lambda_{t-1}\prod_{t-1}^{-1}$ and denoting $S_{t}=V_{t}Q_{t}V_{t}^{T}$, the process update of EKF (Equation (3)) can be written as:

$${\bar{\sum }}_{t}=G_{t}\sum_{t-1}G_{t}^{T}+S_{t}=G_{t}\Lambda_{t-1}\prod_{t-1}^{-1}G_{t}^{T}+S_{t}=G_{t}\Lambda_{t-1}{\left(G_{t}^{-T}\prod_{t-1}\right)}^{-1}+S_{t}$$

Following Equation (5), we have:

(7) $${\bar{\sum }}_{t}={\left((G_{t}^{-t}\prod_{t-1}){\left(G_{t}\Lambda_{t-1}+S_{t}G_{t}^{-T}\prod_{t-1}\right)}^{-1}\right)}^{-1}={\left({\bar{E}}_{t}{\bar{F}}_{t}^{-1}\right)}^{-1}={\bar{F}}_{t}{\bar{E}}_{t}^{-1}$$

where ${\bar{E}}_{t}=G_{t}^{-T}\prod_{t-1}$ and ${\bar{F}}_{t}=G_{t}\Lambda_{t-1}+S_{t}G_{t}^{-T}\prod_{t-1}$,

For computation considerations, the covariance update of the measurement update step can be given by the information form [11]:

(8) $$\begin{array}{c} {\bar{\Omega }}_{t}={\bar{\text{∑}}}_{t}^{-1}={\left(G_{t}\Omega_{t-1}^{-1}+T_{t}\right)}^{-1}\\ \Omega_{t}={\bar{\Omega }}_{t}+H_{t}^{T}R_{t}^{-1}H_{t} \end{array}$$

Denoting $N_{t}=H_{t}^{T}R_{t}^{-1}R_{t}^{-1}H_{t}$, Equation (8) can be rewritten as Ω = Ω̄*_t_*+*N_t_*. Therefore, from Equations (7) and (8), we can write:

$$\sum_{t}\Omega_{t}^{-1}={\left({\bar{\text{∑}}}_{t}^{-1}+N_{t}\right)}^{-1}={\left({\bar{E}}_{t}{\bar{E}}_{t}^{-1}+N_{t}\right)}^{-1}$$

Following Equation (5),

$$\sum_{t}={\bar{F}}_{t}{\left({\bar{E}}_{t}+N_{t}\bar{F}\right)}^{-1}=\Lambda_{t}\prod_{t}^{-1}$$

where:

(9) $$\begin{array}{ll} \Lambda_{t} & ={\bar{F}}_{t}=G_{t}\Lambda_{t-1}+S_{t}G_{t}^{-T}\prod_{t-1}\\ \prod_{t} & ={\bar{E}}_{t}+N_{t}{\bar{F}}_{t}=G_{t}^{-T}\prod_{t-1}+N_{t}(G_{t}\Lambda_{t-1}+S_{t}G_{t}^{-T}\prod_{t-1})\\ & =N_{t}G_{t}\Lambda_{t-1}+(I+N_{t}S_{t})G_{t}^{-T}\prod_{t1} \end{array}$$

As can be seen from Equation (9), Λ*_t_*, П*_t_* are both linear functions of Λ*_t_*_−1_, П*_t_*_−1_.

Denoting Ψ as the stacked block matrix of Λ and П:

(10) $$\Psi =\left[\begin{array}{c} \Lambda \\ \prod \end{array}\right]$$

Equation (10) can be rewritten as:

(11) $$\Psi_{t}=\left[\begin{array}{c} \Lambda_{t}\\ \prod_{t} \end{array}\right]={\left[\begin{array}{cc} 0 & I\\ I & N \end{array}\right]}_{t}{\left[\begin{array}{cc} 0 & G^{-T}\\ G & SG^{-T} \end{array}\right]}_{t}\left[\begin{array}{c} \Lambda_{t-1}\\ \prod_{t-1} \end{array}\right]=\varsigma_{t}\Psi_{t-1}$$

where:

(12) $$\varsigma_{t}=\begin{array}{ccc} {\left[\begin{array}{cc} 0 & I\\ I & N \end{array}\right]}_{t} & {\left[\begin{array}{cc} 0 & G^{-T}\\ G & SG^{-T} \end{array}\right]}_{t}= & {\left[\begin{array}{cc} G & SG^{-T}\\ NG & G^{-T}+NSG^{-T} \end{array}\right]}_{t} \end{array}$$

is the one-step transfer function matrix. Therefore, the covariance at *t* can be recovered using the factors of Ψ*_t_*:

(13) $$\sum_{t}=\Lambda_{t}\prod_{t}^{-1}$$

**Figure 1.**
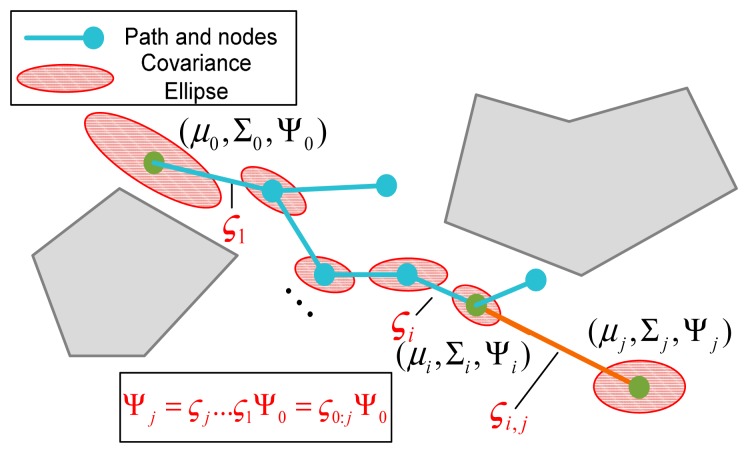
Linear covariance prediction procedure using the transfer function.

Using the above factorization and the linear transfer function, the non-linear Kalman update process of the covariance can be transformed into a linear propagation step of the covariance factors, and the multiple updates can be combined into a single transfer step. This allows for efficient covariance propagation and uncertainty prediction for the sampling-based motion planning strategy: The posterior covariance Σ*_T_* resulting from any initial belief (*μ_t_, Σ_t_*) along the trajectory can be recovered by multiplying multiple transfer functions:

(14) $$\Psi_{T}=\varsigma_{T}\cdots \varsigma_{t+1}\Psi_{t}=\varsigma_{T-t}\Psi_{t}$$

and in a sampling-based path planner that represents paths in the form of trees and nodes, for any arbitrary node *n_j_* in the trajectory, the posterior covariance of the state at the node can be propagated from the predecessor node *j*–*1* along the path in one efficient step using the transfer function *ς*0*_:j_* (Figure 1):

(15) $$\Psi_{j}=\varsigma_{T}\cdots \varsigma_{1}\Psi_{0}=\varsigma_{0:j}\Psi_{0}$$

Taking advantage of the above linear covariance propagation approach, the state estimation covariance of one point *n_i_* (node or time step) can be predicted using the covariance of its adjacent point, as well as the EKF Jacobian matrix set associated with the trajectory between *n_i_*_−1_ and *n_i_*; this procedure can be given as follows:

Step 1: Extract the covariance factors Λ*_i_*_−1_ and П*_i_*_−1_ from block matrix Ψ*_i_*_−1_ of *n_i_*_−1_.
Step 2: Compute the EKF matrix set (*S_i_*, *G_i_* and *N_i_*) based on the state transition model and observation model, as well as the predicted state and simulated observation (Equation (1)).
Step 3: Compute the transfer function ς*_i_* using *S_i_*, *G_i_* and *N_i_*, according to Equation (12).
Step 4: Propagate the block matrix Ψ*_i_* of *n_i_* based on ς*_i_* and Ψ*_i_*_−1_, according to Equation (11), then extract the covariance factors Λ*_i_* and П*_i_* from block matrix Ψ*_i_*.
Step 5: Recover the covariance Σ*_i_* of *n_i_* using Λ*_i_* and П*_i_*: $\sum_{i}=\Lambda_{i}\prod_{i}^{-1}.$.

This covariance propagation procedure allows the path planning algorithm to incorporate the covariance-based qualification of state estimation uncertainty into the planning progress in an efficient way. In the CL-RBT algorithm, which is described in detail in the following section, the state estimation uncertainty is qualified using the trace of the covariance:

(16) $$J\left({\text{∑}}_{i}\right)=tr({\text{∑}}_{i})$$

## 5. Closed-Loop Random Belief Trees Algorithm (CL-RBT)

### 5.1. Data Structure

Similar to the conventional RRT, the CL-RBT operates by constructing a tree of trajectories in the state space (more precisely, the belief space). The tree is defined by nodes and edges that connect different nodes. The primary information stored in a node *n* consists of the mean of the state, uncertainty qualification, the path cost and the parent node:

$$n_{i}≜\left\{\mu_{i},{\text{∑}}_{i},\Psi_{i},J_{i}(\text{∑}),C(n_{i}),n_{i}.\text{parent}\right\}$$

where *μ_i_* and Σ*_i_* represent the mean and the covariance of the state distribution, respectively. Ψ*_i_* is the factored form of the covariance. *J_i_*(Σ) qualifies the uncertainty along the trajectory form the root node to node *n_i_*, which can be calculated using the covariance propagation process described in Section 4: *J_i_*(Σ) = *tr*(Σ*_i_*); *C*(*n_i_*) stores the data of the path execution cost associated with node *n_i_*, which can be split into two parts:

$$C_{i}=\omega_{1}c(n_{i}|n_{root})+\omega_{1}\hat{c}(x_{\text{goal}}|n_{i})$$

where *c*(*n_i_*|*n_root_*) denotes the accumulated execution cost resulting from following the trajectory form tree root *n_root_* to *n_i_*, and *ĉ*(*x_goal_*|*n_i_*) is the estimated cost from *n_i_* to the goal state *x_goal_* (cost-to-go). ω_1_, ω_2_ ∈ [0,1] are weight factors. *n_i_*.parent represents the index for the parent node of *n_i_*.

Every two neighbor nodes (*n_i_*, *n_j_*) in the tree is connected by an edge *e*(*n_i_*, *n_j_*), which represents the closed-loop prediction trajectory {*x_i_*…*x_j_*} and control policy to steer the vehicle from *x_i_* to *x_j_*.

## 5.2. Tree Expansion

The CL-RBT motion planning strategy is built upon the standard closed-loop RRT (CL-RRT) originally proposed by [9] and later extended in [17,18]. Similar to the CL-RRT, our CL-RBT algorithm can be split into two primary processes: a tree expansion process that explores the environment by growing the tree and a path selection and execution loop that selects and executes the optimal portion of the tree. Details of the tree expansion process are described in Algorithm 1.

Unlike the standard RRT that generates candidate control inputs randomly or from a look-up table, the tree expansion process of the CL-RBT predicts the feasible trajectory by incorporating a closed-loop system model consisting of the trajectory-following controller and the vehicle dynamics model (Figure 1). Once the *n_near_* is determined, the planner generates a reference r̂ input (Line 7) using *n_near_* and *x_samp_*; then *r̂* is sent to the closed-loop system consisting of the controller and the vehicle dynamics model: the controller generates the control input*u^* (Line 8), which is then sent to the vehicle dynamics to predict the state output *x̂* by forward simulation (Line 9). The predicted trajectory is then checked against environmental constraints (e.g., collision avoidance) to ensure feasibility.

The CL-RBT algorithm extends the CL-RRT tree expansion by incorporating the prediction of the state (position) uncertainty in growing the tree, using the EKF estimator and the covariance propagation approach (Section 4). First, a node *x_samp_* ∈ *X_free_* is generated by random sampling in the free space (Line 1). After that, the prediction of state estimation uncertainty is incorporated as heuristic information in the nearest-node selection: The nearest node *n_near_* to *x_samp_* by a certain metric is determined from the current tree using the following hybrid heuristic information:

(17) $$i^{*}=\underset{i}{\arg\;\min}\left(\lambda_{1}c(n_{i}|n_{\text{root}})+\lambda_{2}\hat{c}(x_{\text{samp}}|n_{i})+\lambda_{3}\frac{x_{\text{samp}}.{\hat{J}}_{\text{samp}}-n_{i}.J_{i}}{n_{i}.J}\right)$$

where *λ*_1_, *λ*_2_, *λ*_3_ are weighting factors for each item. The above heuristic information consists of three components: the exploration heuristic, the optimization heuristic and the uncertainty heuristic.

**Algorithm 1.** Closed-loop random belief tree: tree expansion.
1Take a sample *x* in the reference space
2Find the nearest node set *N_near_* = {*n_near_*_(_*_i_*_)_}, (*i* ≥ 1) of *x_samp_* from tree *T*, using the hybrid heuristic consisting of the path cost and uncertainty qualification
3**for** each node *n_near_* in the nearest node set *N_near_*
4*k* ← 0
5*x̂ t*+*k* ← final state of *N_near_*
6**while**
*x̂* (*t* + *k*) ∈ *χ_free_* (*t* + *k*) and *x̂* (*t* + *k*) has not reached *x_samp_*
**do**
7Generate reference input *r̂* (*t* + *k*) from *n_near_* to *x_samp_*
8Generate control input *û* (*t* + *k*) from feedback control law
9Simulate *x̂* (*t* + *k*) from state propagation model
10*k* ← *k* + 1
11**end while**
12Generate the predicted trajectory *x̄*(*t*), *t* ∈[*t*_0,_*t*_1_]
13Split the predicted trajectory *x̄*(*t*) and add intermediate nodes *n_i_*
14**if**
*x̄*(*t*) ∈ *X^free^* , ∀*t* ∈[*t*_0,_*t*_1_] **then**
15Add *x_sample_* and all intermediate nodes to ***T****_t_*, **break**
16**else if** all intermediate nodes are feasible
17Add intermediate nodes to *T_t_*, **break**
18**end if**
19**end for**
20**for** each newly added feasible node *n*
**do**
21Update path execution cost estimates for *n*
**(Algorithm 2)**
22simulate the observations **z̄**(*t*) and transfer function along the feasible trajectory between *n* and *n.*parent
23Update the matrix Ψ*_n_* and posterior covariance Σ*_n_* and uncertainty cost *J*(Σ) of *n*
24Update the total cost of *n*
25**end for**


The exploration heuristic *ĉ*(*x_samp_*|*n_i_*) denotes the estimated cost by connecting *n_i_* to the sample *x_samp_*, which enables the path planning algorithm to focus on adding new nodes to the tree and quickly exploring the environment.

The optimization heuristic *c*(*n_i_*|*n_root_*) denotes the accumulated cost of the path from the root node *n_root_* to the candidate node *n_i_*. The purpose of using this optimization heuristic is to bias the tree growth towards paths that reduce the overall accumulated cost.

In order to incorporate the state estimation uncertainty factor into the path planning progress, the CL-RBT adds the uncertainty heuristic in the nearest node selection of the tree expansion. This uncertainty heuristic enables the motion planner to select the node that leads to the lowest posterior state estimation uncertainty if it is connected to *x_samp_*, using the predicted state covariance based on the simulated closed-loop state output and observations along the trajectory between candidate nodes and *x_samp_*. This is realized by qualifying the posterior uncertainty along the path from *n_i_* and *x_samp_* using the linear covariance propagation described in Section 7: given the covariance Σ*_i_* and the associated factored matrix Ψ*_i_* of *n_i_*, the observations **z***_i:samp_* and state output **x̂***_i:samp_* are predicted first based on the closed-loop system model. After that, the matrix set *S_i:samp_*, *G_i:samp_*, *N_i:samp_* and the transfer function ς*_i:samp_* along the path are approximated. Therefore, the posterior covariance Σ̂*_samp_* of *x_samp_* resulting from moving the MAV along the path between *n_i_* and *x_samp_* can be propagated by:

$$\begin{array}{c} {\hat{\Psi }}_{\text{samp}}=\varsigma_{i:\text{samp}}\Psi_{i}={\left[\begin{array}{cc} 0 & I\\ I & N \end{array}\right]}_{i:\text{samp}}{\left[\begin{array}{cc} 0 & G^{-T}\\ G & SG^{-T} \end{array}\right]}_{i:\text{samp}}\left[\begin{array}{c} \Lambda_{i}\\ \prod_{i} \end{array}\right]=\left[\begin{array}{c} {\hat{\Lambda }}_{\text{samp}}\\ {\hat{\text{∏}}}_{\text{samp}} \end{array}\right]\\ {\hat{\text{∑}}}_{\text{samp}}={\hat{\Lambda }}_{\text{samp}}{\hat{\text{∏}}}_{\text{samp}}^{-1} \end{array}$$

hence, the resulting uncertainty cost of *x_samp_* can be given by *x_samp_*.*Ĵ_samp_*=*tr*(Σ̂*_samp_*(.

The tree expansion process may yield one or more candidate feasible nodes and corresponding trajectories. For each candidate node *n* and trajectory, the planner calculates the path cost (Line 21), simulates the observation along the trajectory (Line 22) and, then, further computes the posterior covariance at *n*, as well as the uncertainty qualification of *n* (Line 23). After updating the total cost, the algorithm finally adds the feasible nodes to the current tree *T* (Line 24).

### 5.3. Path Cost Evaluation

As aforementioned in Algorithm 1, once a feasible node is identified and added to the tree, the CL-RRT algorithm evaluates and updates its associated cost (Lines 21–24). In order to address the multiple requirements of operating in complex, GPS-denied environments, the CL-RBT adopts multiple cost metrics that incorporate various factors into these tasks. In CL-RBT, the cost of each node can be divided into two primary categories: the path execution cost and uncertainty cost.

The path execution cost denotes the cost of moving the MAV along the trajectory from the root node *n_root_* to the goal region via the specific node *n_i_*, and it can be given as the following form:

$$C_{p}(n_{i})=\int_{t_{0}}^{t_{i}}c\left[x(t),u(t)\right]dt$$

where [*t*_0_, *t_i_*] is the time interval of the corresponding path (node sequence) {*n_root_*…*n_i_*}, and *c*[*x*(*t*), *u*(*t*)] is the cost metric of the node trajectory (*i.e.*, Euclidian distance, Dubins distance, *etc.*). In the CL-RBT, the path execution cost consists of two primary components: the accumulated path execution cost and the cost-to-go:

$$C_{p}(n_{i})=C(n_{i}|n_{\text{root}}){+}_{\text{CostToGo}}$$

where *C*(*n_i_*|*n_root_*) represents the accumulated execution cost resulting from following the trajectory from *n_root_* to *n_i_*. There are two types of cost estimates of the cost-to-go for each node: a lower bound *C_LB_* and an upper bound *C_UB_*. The lower bound cost-to-go can be given as the distance metric between the MAV's state at the node and the goal state:

$$C_{LB}(n_{i})=C(x_{\text{goal}}|n_{i})$$

The update of the upper bound cost-to-go can be described as follows: each time a new feasible node *n_i_* is added to the tree, the CL-RBT algorithm attempts to connect *n_i_* to the goal by predicting the closed-loop trajectory between *n_i_* and *x_goal_*. If the trajectory is feasible, the upper bound cost-to-go of *n_i_* can be given as the cost associated with this trajectory, otherwise the upper bound cost-to-go of *n_i_* is set to ∞:

$$C_{UB}=\{\begin{array}{c} \int_{n_{j}}^{x_{\text{goal}}}c(x(t),u(t)):\text{feasible trajectory between}\;n_{j},x_{\text{goal}}\;\text{exists}\\ \infty :\text{no feasible trajectory between}\;n_{j},x_{\text{goal}} \end{array}$$

After that, the CL-RBT propagates the paths back towards the root node to update the upper bound cost-to-go of *n_i_*'s affected ancestor nodes: the old upper bound cost-to-go of the parent node is compared with the cost following the newly generated feasible trajectory. If the latter is smaller, the upper bound cost-to-go of the parent node is updated; otherwise, the propagation procedure stops, since there exists a sub-path of the parent with a lower cost-to-go. This update procedure repeats until the current root node is reached. The details of the cost update procedure are shown in Algorithm 2.

**Algorithm 2.** Closed-loop random belief tree: cost update.
1n*_i_*.*C_LB_* ←*c*(*x_goal_*|*n_i_*)
2Compute the accumulated cost of the trajectory between *n_root_* and *n_i_*: *C*(*n_i_*| *n_root_*)
3Generate the trajectory from *n_i_* to goal state *x̄*(*x_goal_* |*n_i_*), using the closed-loop system model
4Check the feasibility of the closed-loop trajectory *x̄*(*x_goal_* |*n_i_*)
5**if**
*x̄*(*x_goal_* |*n_i_*) is feasible **then**
6TrajctiryToGoal← **True**
7*n_i_ C_UB_* ← *C*(*x̄*(*x_goal_*|*n_i_*))(the path cost of *x̄*(*x_goal_* |*n_i_*))
8*n_k_* ←*n_i_*
9**while**
*n_k_* ≠*n_root_*
**and**
*n_k_* .parent.*C_UB_* >*n_k_*.*C_UB_* + *C*(*n_k_*.parent |*n_k_*)
10*n_k_*.parent.*C_UB_* ← *n_k_*.*C_UB_* + *C*(*n_k_*.parent |*n_k_*)
11*n_k_* ← *n_k_*.parent
12**end while**
13*n_i_*.*C*_CostToGo_ ←*n_i_*.*C_BU_*
14**else**
15TrajctoryToGoal← **False**
16*n_i_*.*C_UB_* ← ∞
16*n_i_*.*C*_CostToGo_ ←*n*.*C_LB_*
17**end if**
18*n_i_*.*C* ←*C*(*n_i_*|*n_root_*)+*n_i_*. *C*_CostToGo_
19**return** TrajctoryToGoal


Once a feasible trajectory to the goal is identified, the upper bound cost-to-go is used as the estimated cost-to-go of *n_i_*; otherwise, the lower bound cost-to-go is used. Therefore, the overall path execution cost of node *n_i_* can be given as:

(18) $$C(n_{i})=C(n_{i}|n_{root})+C_{\text{CostToGo}}=C(n_{i}|n_{root})+\{\begin{array}{c} C_{UB}:\text{TrajectoryToGoal}=\text{True}\\ C_{LB}:\text{TrajectoryToGoal}=\text{False} \end{array}$$

(Note that the cost update procedure also identifies feasible paths that connect the goal to the current tree. This enables the CL-RRT to quickly find paths that reach the goal. As the tree grows, more paths to the goal can be found).

The CL-RBT incorporates the qualification of state estimation uncertainty in the cost function of nodes, such that the generated paths ensure accurate state estimation. As discussed previously, the state estimation uncertainty is evaluated using the uncertainty cost, e.g., the trace of the predicted covariance: *J*(n*_i_*) =*tr* (Σ*_ni_*) . As shown in Algorithm 1, once a feasible node is added to the tree and its path execution cost is updated, the CL-RBT predicts its posterior state covariance through the update steps described in Section 4 and computes its uncertainty cost (Algorithm 1, Lines 22, 23). Taking the path execution cost and uncertainty cost into consideration, in CL-RBT, the following multi-objective cost function is used:

(19) $$C(n_{i})=\zeta_{1}C\left(n_{i}|n_{\text{root}}\right)+\zeta_{2}C_{\text{CostToGo}}+\zeta_{3}J(n_{i}.\text{∑})$$

where *C*_CostToGo_ denotes the estimated cost-to-go from *n_i_* to the goal state, *C*(*n_i_*|*n_root_*) is accumulated along the path from root to *n_i_*, and the first two items correspond to the path-execution cost. *J*(*n_i_*.Σ) is the cost associated with the uncertainty of node *n_i_*, which represents the state uncertainty resulting from following path {*n_root_*…*n_i_*}. *ζ*_1_, *ζ*_2_, *ζ*_3_ are weight factors for adjusting the relative importance of finding a short duration path and reducing state estimation uncertainty.

Since there is a unique path from the root to each of the nodes in the tree, the cost of a node can be used to represent the cost of the associated trajectory. The total cost of the node is used to identify the current best trajectory for execution in the selection and execution procedure, which is presented in the next section.

### 5.4. Path Selection and Execution

In order to account for the changes in time-varying operating environments, the motion planner operates by periodically selecting and executing the best portion of the current trajectory while continuously expanding the tree during the execution process. The path selection and execution process is presented in Algorithm 3.

Denote the period for the path selection and execution as Δ*t*. At the beginning of each iteration period, the algorithm updates the current actual state of the MAV, as well as the environment information. The purpose of this step is to ensure the MAV's situational awareness (Line 4). After that, the state is first propagated to the end of the period, resulting in *x̄*(*t*_0_ + Δ*t*) (Line 4). Then, the current root of the tree is set to the most current node that is followed by the propagated state, and all other children nodes of the previous root are removed. This is because the MAV is considered to have passed the previous root, and the paths of its children nodes will never be executed. During each of the iterations, the planner identifies the best portion of the trajectory in terms of the cost metric. Since each node *n_i_* in the tree is associated with a single path, the trajectory can be uniquely specified using the sequence consisting of all of the nodes from root to *n_i_*:{*n_root_*…*n_i_*}. In CL-RBT, the cost function given in Equation (19) is used for selecting the current best paths. This multi-objective cost metric enables the motion planner to select paths that achieve a balance between finding low-cost paths to the goal and minimizing localization uncertainty. At the end of each path selection iteration step, the selected feasible trajectory is sent to the controller to be executed, while the tree keeps expanding (Algorithm 1) during the remaining time of period Δ*t*. This mechanism guarantees that the path planning algorithm can provide a feasible path for execution in real time and account for uncertainty in a dynamic environment.

**Algorithm 3.** Closed-loop random belief tree: execution loop.
1*t* ← 0
2Initialize tree *T* from node at *x_initial_*
3**while**
*x*(*t*) *x*(*t*) ∉ *X_goal_*
**do**
4Update the current actual state *x*(*t*_0_) and environment information
5Predict state *x*(*t*) to *x̄*(*t*_0_ + Δ*t*) and simulate observations
6**while** time remaining < Δ*t*
**do**
7expand the tree using Algorithm1
8**end while**
9Select the current best feasible path (node sequence) P*={*n*_0_…*n_k_*} according to the multiple-objective cost function given as Equation (19)
10**if** no best feasible path exists **then**
11Send brake command to the controller and **goto** 3
12**else**
13Send *p* * to controller to execute
14**end if**
15*t* ← *t* + Δ*t*
16**end while**


**Figure 2.**
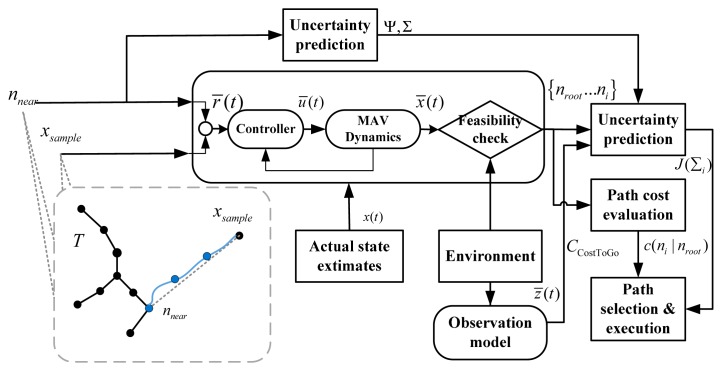
Diagram of the closed-loop random belief tree motion planning framework. MAV, micro-aerial vehicle.

The overall diagram of the CL-RBT algorithm is depicted in Figure 2.

## 6. Indoor Flight Simulation Results Based on a Simulated Laser Scanner-Equipped MAV

In order to validate the effectiveness of the CL-RBT motion planner, we have conducted a number of experiments in various simulated scenarios that are derived from real-world environments. The first scenario consists of a quadrotor MAV navigating in a 3D wide-open indoor environment, with all structures that can be detected by laser rangefinders concentrated along the peripheral walls. The first scenario is used to demonstrate the CL-RBT's ability to generate the path that ensures the MAV's localization confidence. The subsequent scenarios involve more complex extensions, including 3D unstructured environments (the second scenario), and the purpose of these scenarios is to validate the effectiveness of the CL-RBT algorithm in generating paths that satisfy multiple constraints imposed by dynamic, unstructured environments and non-linear MAV dynamics, while achieving a trade-off between minimizing path cost and reducing localization uncertainty in GPS-denied environments.

For all of these experiments, we assume that the dynamics model of the quadrotor is non-linear and that the environment is without access to GPS signals. The quadrotor must start from an initial spot and traverse through the obstacles to a goal location, relying on a path-following control law and the state estimates provided by an EKF estimator. The exteroceptive sensor equipped on the quadrotor is assumed to be a simulated laser rangefinder with a maximum sensing range of 2 m and a 240° field-of-view. For each of these environments, a 3D model of the environment is first generated using an RGB-D-based environmental modeling approach from our previous work [26]. The 3D point-cloud model is then transformed into a polyhedron-based model, which is used for path planning. The CL-RBT is implemented in MATLAB^®^ and all simulations are performed in real time on an Intel 3.2 GHz platform with a 4 G RAM. The CL-RBT algorithm selects and executes the best parts of the paths every Δ*t* seconds.

### 6.1. Quadrotor Dynamics Model

**Figure 3.**
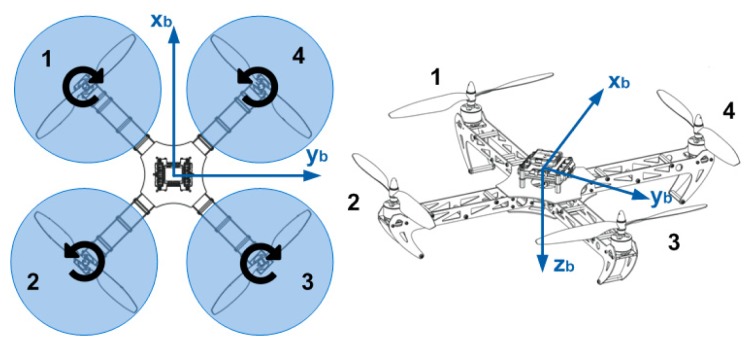
Structure and configuration of the quadrotor MAV.

The quadrotor model that is used in the simulation is depicted in Figure 3. For the simulation experiments, the x-y-z body-fixed coordinates of the quadrotor is derived using the square configuration, and the kinematic and dynamic model of the quadrotor is developed as rigid-body dynamics influenced by the gravity and thrust of the rotors, assuming near-hover aerodynamics and neglecting the effects caused by the translational velocity. The pure pursuit reference law [27] is applied to steer the reference trajectory, and a PD control law is used to control the quadrotor tracking of the reference. This closed-loop system model consisting of the dynamics model and the control law is used for both the trajectory prediction of the CL-RBT planner and the execution of the resultant trajectory.

The configuration of the quadrotor utilized in this paper is illustrated in Figure 3. Two coordinate systems are considered in our scheme: the navigation frame (Earth-fixed frame) (**x***_E_*, **y***_E_*, **z***_E_*) and the body-fixed frame (**x***_b_*, **y***_b_*, **z***_b_*). The translational and rotational motions of the quadrotors are achieved through the forces and moments caused by varying the angular rates of the four propellers. Assuming that the quadrotor structure is rigid and symmetrical and the center of mass coincides with the origin of the body-fixed frame, the equations of motion of the quadrotor can be derived using Newton's law and Euler equations [27]. Define *φ*, *θ*, *ψ* as the Euler angles denoting the roll, pitch and yaw attitude, respectively, the rotational dynamics can be described as:

(20) $$\begin{array}{l} \ddot{\varphi }=\dot{\theta }\dot{\psi }\left(\frac{I_{yy}-I_{zz}}{I_{xx}}\right)+\frac{l}{I_{xx}}b(\omega_{3}^{2}+\omega_{4}^{2}-\omega_{1}^{2}-\omega_{2}^{2})-\frac{K_{drk}}{I_{xx}}\dot{\varphi }+\frac{J_{r}}{I_{xx}}\dot{\theta }(\omega_{2}+\omega_{4}-\omega_{1}-\omega_{3})\\ \ddot{\theta }=\dot{\varphi }\dot{\psi }\left(\frac{I_{zz}-I_{xx}}{I_{yy}}\right)+\frac{l}{I_{yy}}b(\omega_{2}^{2}+\omega_{3}^{2}-\omega_{1}^{2}-\omega_{4}^{2})-\frac{K_{dry}}{I_{yy}}\dot{\theta }-\frac{J_{r}}{I_{yy}}\dot{\varphi }(\omega_{2}+\omega_{4}-\omega_{1}-\omega_{3})\\ \ddot{\psi }=\dot{\varphi }\dot{\theta }\left(\frac{I_{xx}-I_{yy}}{I_{zz}}\right)+\frac{1}{I_{zz}}d(\omega_{1}^{2}+\omega_{3}^{2}-\omega_{4}^{2}-\omega_{2}^{2})-\frac{K_{drz}}{I_{zz}}\dot{\psi } \end{array}$$

where *I_xx_*, *I_yy_*, *I_zz_* are the moments of inertial around the three axis, *J_r_* is the rotor inertia and *l* denotes the length of the moment arm. *ω*_1_, *ω*_2_, *ω*_3_, *ω*_4_ are the four propellers' rotation speeds. *b* and *d* denote the rotor thrust coefficient and rotor drag coefficient, respectively. *K_drx_*, *K_dry_*, *K_drz_* represent the rotational drag coefficients of the quadrotor body.

Define (*x*, *y*, *z*) as the three-dimensional position with respect to the navigation frame; the translational dynamics of the quadrotor can be given as:

(21) $$\begin{array}{l} ẍ=(\cos\varphi \cos\psi \sin\theta +\sin\varphi \sin\psi )\frac{1}{m}\overset{4}{\underset{i=1}{\text{∑}}}T_{i}-\frac{K_{\text{dtx}}}{m}ẋ\\ ÿ=(\sin\psi \sin\theta \cos\varphi -\sin\varphi \cos\psi )\frac{1}{m}\overset{4}{\underset{i=1}{\text{∑}}}T_{i}-\frac{K_{\text{dty}}}{m}ẏ\\ \ddot{z}=\cos\theta \cos\varphi \frac{1}{m}\overset{4}{\underset{i=1}{\text{∑}}}T_{i}-\frac{K_{\text{dtz}}}{m}ż-g \end{array}$$

where *T_i_* denotes the thrust generated by propeller *i* and $T_{i}=b\omega_{i}^{2}$, m is the total mass of the quadrotor and *g* represents the gravitational acceleration. *K_drx_*, *K_dry_*, *K_drz_* are the translational drag coefficients of the quadrotor body.

### 6.2. Control Scheme Design of the MAV Model

#### 6.2.1. Cascaded Control Scheme

Under the assumption of near-hovering velocities, the drag terms caused by the translational and rotational motions in Equations (20) and (21) can be neglected, and the system dynamics model can be described in the following state-space form (**ẋ** = *f*(**x,u**)):

(22) $$\dot{\text{x}}=\left[\begin{array}{c} \dot{\varphi }\\ \dot{\theta }\\ \dot{\psi }\\ \dot{\theta }\dot{\psi }\left(\frac{I_{yy}-I_{zz}}{I_{xx}}\right)+\frac{l}{I_{xx}}bu_{2}\\ \dot{\varphi }\dot{\psi }\left(\frac{I_{zz}-I_{xx}}{I_{yy}}\right)++\frac{l}{I_{yy}}bu_{3}\\ \dot{\varphi }\dot{\theta }\left(\frac{I_{xx}-I_{yy}}{I_{zz}}\right)+\frac{l}{I_{zz}}du_{4}\\ ẋ\\ ẏ\\ ż\\ (\cos\varphi \cos\psi \sin\theta +\sin\varphi \sin\psi )\frac{1}{m}u_{1}\\ (\sin\psi \sin\theta \cos\varphi -\sin\varphi \cos\psi )\frac{1}{m}u_{1}\\ \cos\theta \cos\varphi \frac{1}{m}u_{1}-g \end{array}\right]$$

where **x** = [*φ θ ψ φ̇ θ̇ ψ̇ x y z ẋ ẏ ż*] *^T^* denotes the state vector, and **u** =[*u*_1_ *u*_2_ *u*_3_ *u*_4_]*^T^* represents the control input:

(23) $$\text{u}=\left[\begin{array}{c} b(\omega_{1}^{2}+\omega_{2}^{2}+\omega_{3}^{2}+\omega_{4}^{2})\\ b(\omega_{3}^{2}+\omega_{4}^{2}-\omega_{1}^{2}-\omega_{2}^{2})\\ b(\omega_{2}^{2}+\omega_{3}^{2}-\omega_{1}^{2}-\omega_{4}^{2})\\ d(\omega_{1}^{2}+\omega_{3}^{2}-\omega_{4}^{2}-\omega_{2}^{2}) \end{array}\right]$$

The physical meaning of the control input is related to the forces generated by the four propellers: *u*_1_ represents the total thrust of the propellers, while *u*_2_, *u*_3_ and *u*_4_ are the differences of the propeller pairs.

As can be seen from Equation (20), the rotational dynamics (*φ*, *θ*, *ψ*) are independent of the translational dynamics (*x*, *y*, *z*), leading to decoupled system dynamics. In addition, the altitude dynamics (z) can also be decoupled from the planar translational dynamics (*x*, *y*) by assuming that the *φ*, *θ* angles are very small. As a result, the quadrotor dynamics in Equation (21) can be decoupled into independent sub-system dynamics, and the cascaded control systems for the attitude, position and altitude can be designed from the decoupled dynamics. Under the assumption of small *φ*, *θ* angles and near-hovering motions, we can derive the following linearized attitude dynamics:

(24) $$\{\begin{array}{c} \ddot{\varphi }=\frac{l}{I_{x}}bu_{2}\\ \ddot{\theta }=\frac{l}{I_{y}}bu_{3}\\ \ddot{\psi }=\frac{d}{I_{z}}u_{4} \end{array}$$

The linearized translational motion dynamics can be given as:

(25) $$\{\begin{array}{l} ẍ=(\theta \cos\psi +\varphi \sin\psi )\frac{1}{m}u_{1}\\ ÿ=(\theta \sin\psi -\varphi \cos\psi )\frac{1}{m}u_{1}\\ \ddot{z}=\frac{1}{m}u_{1}-g \end{array}$$

**Figure 4.**
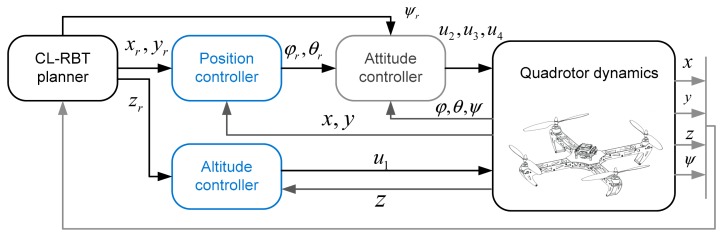
Cascaded control scheme of the quadrotor MAV. CL-RBT, closed-loop random belief trees.

Using the decoupled rotational and translational dynamics in Equations (24) and (25), we can design a cascaded control scheme consisting of the inner control loop and outer control loop, of which the control laws can be designed separately. As illustrated in Figure 4, the outer control loop is the position and altitude controller, which receives the reference path from the CL-RBT planner and generates the reference roll and pitch commands (*φ_r_*, *θ_r_*) to the inner attitude control loop, while the attitude controller produces the desired rotor speed to achieve the reference attitude according to the attitude commands *φ_r_*, *θ_r_*, *ψ_r_*.

#### 6.2.2. Controller Design

Based on the attitude and translational motion dynamics described in Equations (24) and (25), the proportional-integral-derivative (PID) control scheme is utilized to design the attitude and position controller for the quadrotor MAV. The first step is to consider the inner loop controller, which functions as the core of the control scheme. Using the attitude dynamics shown in Equation (24), the attitude controller can be designed as follows:

(26) $$\begin{array}{l} u_{2}=K_{P}^{\varphi ,\theta }(\varphi_{r}-\varphi )+K_{I}^{\varphi ,\theta }\int (\varphi_{r}-\varphi )dt+K_{D}^{\varphi ,\theta }({\dot{\varphi }}_{r}-\dot{\varphi })\\ u_{3}=K_{P}^{\varphi ,\theta }(\theta_{r}-\theta )+K_{I}^{\varphi ,\theta }\int (\theta_{r}-\theta )dt+K_{D}^{\varphi ,\theta }({\dot{\theta }}_{r}-\dot{\theta })\\ u_{4}=K_{P}^{\psi }(\psi_{r}-\psi )+K_{I}^{\psi }\int \left(\psi_{r}-\psi )dt+K_{D}^{\psi }({\dot{\psi }}_{r}-\dot{\psi }\right) \end{array}$$

where $K_{P}^{\varphi ,\theta }$, $K_{I}^{\varphi ,\theta }$, $K_{D}^{\varphi ,\theta }$, $K_{P}^{\psi }$, $K_{I}^{\psi }$, $K_{D}^{\psi }$ are the control gains of the PID controller and *φ_r_*, *θ_r_* and *ψ_r_* denote the reference roll, pitch and yaw, respectively. Note that *φ_r_*, *θ_r_* are generated by the outer control loop while *ψ_r_* is directly provided by the CL-RBT planner.

Under the assumption of near-hovering velocities and small rotational angle motions, the control scheme of translational dynamics can be decoupled into the planar (*x*, *y*) and altitude controllers that can be designed separately. The altitude controller is designed based on PID control with non-linear compensation:

(27) $$u_{1}=\frac{1}{\cos\theta \cos\varphi }(K_{P}^{z}(z_{r}-z)+K_{I}^{z}\int \left(z_{r}-z)dt+K_{D}^{z}({ż}_{r}-ż\right))$$

where $K_{p}^{z}$, $K_{I}^{z}$, $K_{D}^{z}$ are the PID control gains, and z*_r_* is the reference altitude of the path provided by the CL-RBT planner. The planar position controllers take the following form:

$$\begin{array}{ll} \theta_{r} & =\cos\psi [K_{P}^{x}(x_{r}-x)+K_{I}^{x}\int (x_{r}-x)dt+K_{D}^{x}({ẋ}_{r}-ẋ)]\\ & +\sin\psi [K_{P}^{y}(y_{r}-y)+K_{I}^{y}\int (y_{r}-y)dt+K_{D}^{y}({ẏ}_{r}-ẏ)]\\ \phi_{r} & =\sin\psi [K_{P}^{x}(x_{r}-x)+K_{I}^{x}\int (x_{r}-x)dt+K_{D}^{x}({ẋ}_{r}-ẋ)]\\ & -\cos\psi [K_{P}^{y}(y_{r}-y)+K_{I}^{y}\int (y_{r}-y)dt+K_{D}^{y}({ẏ}_{r}-ẏ)] \end{array}$$

where $K_{p}^{x}$, $K_{I}^{x}$, $K_{D}^{x}$, $K_{p}^{y}$, $K_{I}^{y}$, $K_{D}^{y}$ are the PID control gains and *x_r_*, *y_r_* are the reference position provided by the CL-RBT planner. As mentioned before, the outer loop controller outputs *θ_r_*, *φ_r_* to the inner loop controller as the reference pith and roll, which are used in Equation (26) for attitude control.

In our applications, the frequency of the inner loop (attitude controller) is 250 Hz, while the outer loop (translational and altitude controller) runs at 30 Hz. The PID gains of the controllers are tuned through extensive numerical simulations in order to achieve a desirable control performance.

In this paper, we only report simulation results based on the PID control law. The reason for selecting the PID scheme is that it can handle complex dynamics model and it is robust to modeling errors. Note that more complex and advanced control laws can also be adopted in the CL-RBT framework. In addition, other types of MAV dynamics models with associated guidance and control laws can be incorporated to form the closed-loop model in CL-RBT.

### 6.3. EKF Process Model

In the simulation experiments, the states of the MAV are estimated using an EKF-based estimator. The MAV states to be estimated in the EKF filter include: three-axis position in the global frame **p** = [*x*, *y*, *z*]^T^, MAV orientation represented using Euler angles [*φ, θ, ψ*]^T^ (roll, pitch and yaw), three-axis velocity in the global frame **v** = [*v_x_*, *v_y_*, *v_z_*]^T^, as well as the gyroscope bias **b**_ω_ = [*b*_ω_*_x_*, *b*_ω_*_y_*, *b*_ω_*_z_*]^T^ and accelerometer bias **b***_f_* = [*b_fx_*, *b_fy_*, *b_fz_*]^T^. The estimated state vector can be denoted specifically as follows:

(28) $$\text{x}={\left[\text{p},\text{v},\varphi ,\theta ,\psi ,{\text{b}}_{\omega },{\text{b}}_{f}\right]}^{\text{T}}={\left[x,y,z,v_{x},v_{y},v_{z},\varphi ,\theta ,\psi ,b_{\omega x},b_{\omega y},b_{\omega z},b_{fx},b_{fy},b_{fz}\right]}^{\text{T}}$$

As described previously, the MAV is equipped with a laser rangefinder, which is used by scan-matching to provide position and heading measurements (*x*, *y*, *ψ*). The altitude of the MAV is measured by an onboard sonar altimeter, and it is assumed that the altitude measurement is not affected by the attitude of the MAV. In addition to the laser rangefinder, the MAV is assumed to be equipped with an IMU module, which consists of a triaxial gyroscope and triaxial accelerometer. The triaxial gyroscope provides three-axis angular rates ω*_m_* = [ω*_mx_*, ω*_my_*, ω*_mz_*]^T^, while the accelerometer measures the three-axis accelerations *f_m_* = [*f_mx_*, *f_my_*, *f_mz_*]^T^, and the above IMU measurements are all expressed in the MAV's body frame. Using the above definitions, the continuous state-space-based process model (**x**= *g*(**x,u**)) describing the IMU dynamics can be given by:

(29) ṗ=v

(30) $$\dot{\text{v}}={\tilde{C}}_{b}^{n}(f_{m}-{\text{b}}_{f})-\;\text{g}$$

(31) $$\begin{array}{ccc} \left[\begin{array}{c} \dot{\phi }\\ \dot{\theta }\\ \dot{\psi } \end{array}\right]= & \left[\begin{array}{ccc} 1 & \sin\phi \tan\theta & \cos\phi \tan\theta \\ 0 & \cos\phi & -\sin\phi \\ 0 & \sin\phi \sec\theta & \cos\phi \sec\theta \end{array}\right] & \left[\begin{array}{c} \omega_{mx}-b_{\omega x}\\ \omega_{my}-b_{\omega y}\\ \omega_{mz}-b_{\omega z} \end{array}\right] \end{array}$$

(32) $${\dot{\text{b}}}_{\omega }=0$$

(33) $${\dot{\text{b}}}_{f}=0$$

where ${\tilde{C}}_{b}^{n}$ denotes the transformation matrix from the body frame to the global frame, and **g** = [0, 0, *g*]^T^ is the local gravity vector in the global frame. Since the *x*, *y* position and the heading *ψ* (yaw) of the MAV are estimated separately using the measurements from the laser scan-matching, ψ can be treated independent of roll *φ* and pitch *θ*, and the MAV motion of the *x*-, *y*-axes can be decoupled from *z*-axis motion under the assumption of near-hovering velocity and small *φ*, *θ* angles. This assumption yields the simplified transformation matrix in Equation (30):

(34) $${\tilde{C}}_{b}^{n}=\left[\begin{array}{ccc} \cos\psi & -\sin\psi & 0\\ \sin\psi & \cos\psi & 0\\ 0 & 0 & 1 \end{array}\right]$$

A similar approach for the simplification of the MAV motion can also be found in [28,29] (Note that although this simplified velocity model may cause significantly large estimation error when the MAV performs aggressive attitude maneuvers or high-speed flight, it is sufficiently accurate for state estimation under the above assumptions in this paper).

To implement the state estimation on a computer system, the above process model is transformed into a discrete-time model. Let Δ*t* be the update period of EKF and assume that Δ*t* is sufficiently small, the process model in Equations (29)–(33) can be discretized as follows:

(35) $${\text{x}}_{t}={\text{x}}_{t-1}+\Delta tf({\text{x}}_{t-1},{\text{u}}_{t-1})$$

In order to implement the EKF estimator, the above discrete process model needs to be further linearized by calculating the Jacobians *∂g*/*∂***x**|*x*_t1_ as described in Equation (2). To derive the Jacobians, the calculation of partial derivatives of the process model with respect to the state vector is given as follows:

(36) $$G={\frac{\partial g}{\partial \text{x}}|}_{{\text{x}}_{t-1}}=I+\Delta t{\frac{\partial f}{\partial \text{x}}|}_{{\text{x}}_{t-1}}=\left[\begin{array}{ccccc} I_{3\times 3} & \Delta tI_{3\times 3} & 0_{3\times 3} & 0_{3\times 3} & 0_{3\times 3}\\ 0_{3\times 3} & I_{3\times 3} & \Delta tG_{23} & 0_{3\times 3} & \Delta tG_{25}\\ 0_{3\times 3} & 0_{3\times 3} & I_{3\times 3}+\Delta tG_{33} & \Delta tG_{34} & 0_{3\times 3}\\ 0_{3\times 3} & 0_{3\times 3} & 0_{3\times 3} & I_{3\times 3} & 0_{3\times 3}\\ 0_{3\times 3} & 0_{3\times 3} & 0_{3\times 3} & 0_{3\times 3} & I_{3\times 3} \end{array}\right]$$

where:

$$G_{23}=\left[\begin{array}{ccc} 0 & 0 & -\sin\psi (f_{mx}-b_{fx})-\cos\psi (f_{my}-b_{fy})\\ 0 & 0 & \cos\psi (f_{mx}-b_{fx})-\sin\psi (f_{my}-b_{fy})\\ 0 & 0 & 0 \end{array}\right],G_{25}=\left[\begin{array}{ccc} -\cos\psi & \sin\psi & 0\\ -\sin\psi & -\cos\psi & 0\\ 0 & 0 & -1 \end{array}\right]$$

$$G_{33}=\left[\begin{array}{ccc} (\omega_{my}-b_{\omega y})\cos\varphi \tan\theta -(\omega_{mz}-b_{\omega z})\sin\varphi \tan\theta & (\omega_{my}-b_{\omega y})\sin\varphi \sec^{2}\theta +(\omega_{mz}-b_{\omega z})\cos\varphi \sec^{2}\theta & 0\\ -(\omega_{my}-b_{\omega y})\sin\varphi -(\omega_{mz}-b_{\omega z})\cos\varphi & 0 & 0\\ (\omega_{my}-b_{\omega y})\cos\varphi \sec\theta -(\omega_{mz}-b_{\omega z})\sin\varphi \sec\theta & (\omega_{my}-b_{\omega y})\sin\varphi \sin\theta \sec^{2}\theta +(\omega_{mz}-b_{\omega z})\cos\varphi \sin\theta \sec^{2}\theta & 0 \end{array}\right]$$

$$G_{34}=\left[\begin{array}{ccc} -1 & -\sin\varphi \tan\theta & -\cos\varphi \tan\theta \\ 0 & -\cos\varphi & \sin\varphi \\ 0 & -\sin\varphi \sec\theta & -\cos\varphi \sec\theta \end{array}\right]$$

The process noise *w* is introduced by the IMU angular rates (*ω_m_*) and accelerations (*f*_ω_), which are included as the input **u** to the process model:

(37) $$\text{u}={\left[\omega_{m},f_{m},\right]}^{\text{T}},w={\left[w_{\omega },w_{f}\right]}^{\text{T}}$$

where *w*_ω_, *w_f_* are the noise associated with the angular rates and accelerations, respectively, and *w*_ω_, *w_f_* are both white Gaussian noises with zero mean, *i.e.*, *w*_ω_∼*N*(0, *Q*_ω_), *w_f_*∼*N*(0, *Q_f_*). Similarly, the partial derivatives of the process model with respect to the process noise can be calculated by:

(38) $$V_{t}={\frac{\partial g}{\partial w}|}_{{\text{x}}_{t-1},{\text{u}}_{t-1}}=\Delta t{\frac{\partial f}{\partial w}|}_{{\text{x}}_{t-1},{\text{u}}_{t-1}}={\left[\begin{array}{ccccc} 0_{3\times 3} & 0_{3\times 3} & \Delta tV_{31} & 0_{3\times 3} & 0_{3\times 3}\\ 0_{3\times 3} & \Delta tV_{22} & 0_{3\times 3} & 0_{3\times 3} & 0_{3\times 3} \end{array}\right]}^{\text{T}}$$

where:

$$V_{22}={\tilde{C}}_{b}^{n},V_{31}=\left[\begin{array}{ccc} 1 & \sin\varphi \tan\theta & \cos\varphi \tan\theta \\ 0 & \cos\varphi & -\sin\varphi \\ 0 & \sin\varphi \sec\theta & \cos\varphi \sec\theta \end{array}\right]$$

Due to the different measurement rates of the IMU, the sonar and the scan-matching module, the state and covariance are updated separately at different times, using three groups of measurements. The pitch, roll angle (*φ*, *θ*) and the gyroscope bias **b**_ω_ are updated first using the measurements from the IMU (more specifically, the accelerometer measurements). The altitude *z* is updated separately using the sonar measurement, since it is uncorrelated with the other measurements. In addition, the update of the MAV position **p**, heading *ψ*, the velocity *v_x_*, *v_y_* and the accelerometer bias **b***_f_* are completed when the measurements from laser scan-matching are received. By the above discussions, while the performance of pitch and roll (*φ*, *θ*) estimates depends on the IMU, the estimation of *x*, *y* position and the heading ψ are primarily affected by the characteristics of the laser rangefinder, as well as the perceived environment information. As a result, in order to evaluate how the path planning strategy affects the uncertainty of state estimation, we extract the laser-related states **x̃**=[*x*,*y*,*v_x_*,*v_y_*, *ψ,b_fx_*, *b_fy_*]^T^ from the full state vector. By Equation (35), the process update model of **x̃** can be given as:

(39) $$\begin{array}{l} x_{t}=x_{t-1}+v_{x|t-1}\Delta t\\ y_{t}=y_{t-1}+v_{y|t-1}\Delta t\\ \left[\begin{array}{c} v_{x}\\ v_{y} \end{array}\right]={\left[\begin{array}{c} v_{x}\\ v_{y} \end{array}\right]}_{t-1}+\Delta t{\left[\begin{array}{cc} \cos\psi & -\sin\psi \\ \sin\psi & \cos\psi \end{array}\right]}_{t-1}{\left[\begin{array}{c} f_{mx}-b_{fx}\\ f_{my}-b_{fy} \end{array}\right]}_{t-1}+\Delta t{\left[\begin{array}{cc} \cos\psi & -\sin\psi \\ \sin\psi & \cos\psi \end{array}\right]}_{t-1}\left[\begin{array}{c} w_{fx}\\ w_{fy} \end{array}\right]\\ \psi_{t}=\psi_{t-1}+\Delta t\left(\omega_{mz|t-1}-b_{\omega z|t-1}\right)+\Delta tw_{\omega z}\\ b_{fx|t}=b_{fx|t-1}\\ b_{fy|t}=b_{fy|t-1} \end{array}$$

By the above process model, we have:

(40) $${\tilde{\text{x}}}_{t}=\tilde{g}\left({\tilde{\text{x}}}_{t-1},{\text{ũ}}_{t-1},\tilde{w}\right)$$

where **ũ**=[*f_mx_*,*f_my_*, *ω_mz_*]^T^, *w̃*=[*w_fx_*,*w_fy_*,*w_ωz_*] , and the Jacobians of the above model can be obtained directly from Equations (36) and (38):

(41) $${\tilde{G}}_{t}={\frac{\partial g}{\partial \text{x}}|}_{{\text{x}}_{t-1}{\text{u}}_{t-1}}=\left[\begin{array}{ccccccc} 1 & 0 & \Delta t & 0 & 0 & 0 & 0\\ 0 & 1 & 0 & \Delta t & 0 & 0 & 0\\ 0 & 0 & 1 & 0 & -\Delta t\sin\psi \left(f_{mx}-b_{fx}\right)-\Delta t\cos\psi \left(f_{my}-b_{fy}\right) & -\Delta t\cos\psi & \Delta t\sin\psi \\ 0 & 0 & 0 & 1 & \Delta t\cos\psi \left(f_{mx}-b_{fx}\right)-\Delta t\sin\psi \left(f_{my}-b_{fy}\right) & -\Delta t\sin\psi & -\Delta t\cos\psi \\ 0 & 0 & 0 & 0 & 1 & 0 & 0\\ 0 & 0 & 0 & 0 & 0 & 1 & 0\\ 0 & 0 & 0 & 0 & 0 & 0 & 1 \end{array}\right]$$

(42) $${Ṽ}_{t}=\left[\begin{array}{ccccccc} 0 & 0 & \Delta t\cos\psi & \Delta t\sin\psi & 0 & 0 & 0\\ 0 & 0 & -\Delta t\sin\psi & \Delta t\cos\psi & 0 & 0 & 0\\ 0 & 0 & 0 & 0 & \Delta t & 0 & 0 \end{array}\right]$$

Denote $\sigma_{fx}^{2}$, $\sigma_{fy}^{2}$ and $\sigma_{\omega z}^{2}$ as the noise covariance of the accelerometer measurements in the *x*- and *y*-axis, as well as the gyroscope measurements in the *z*-axis, respectively. The process noise covariance of the process model in Equation (39) can be given by:

(43) $$Q=\text{diag}\left(\sigma_{fx}^{2},\sigma_{fy}^{2},\sigma_{\omega z}^{2}\right)$$

Therefore, the matrix *S_t_* in Equation (11) can be calculated by $S_{t}={Ṽ}_{t}Q{Ṽ}_{t}^{T}$, and *G̃_t_* (Equation (41)), *Ṽ_t_* (Equation (42)), *S_t_* are used in the linear covariance propagation, as described in Equation (11).

### 6.4. Sensor Measurement Model and Uncertainty Analysis of Laser Rangefinders

In many actual applications, a MAV performs localization using an onboard laser rangefinder, which obtains measurements through a series of range readings recorded at consecutive angles increments. Therefore, the accuracy of the localization is mainly affected by the range to the objects and the topology of the environments. In our simulation, we assume that the quadrotor is equipped with a laser rangefinder and that the localization is performed by matching the laser scans. In order to incorporate the uncertainty of the laser rangefinder for the covariance propagation in the CL-RBT planner, we used an information matrix-based method [6] to determine the uncertainty of the laser measurements with respect to the topology of the environments.

**Figure 5.**
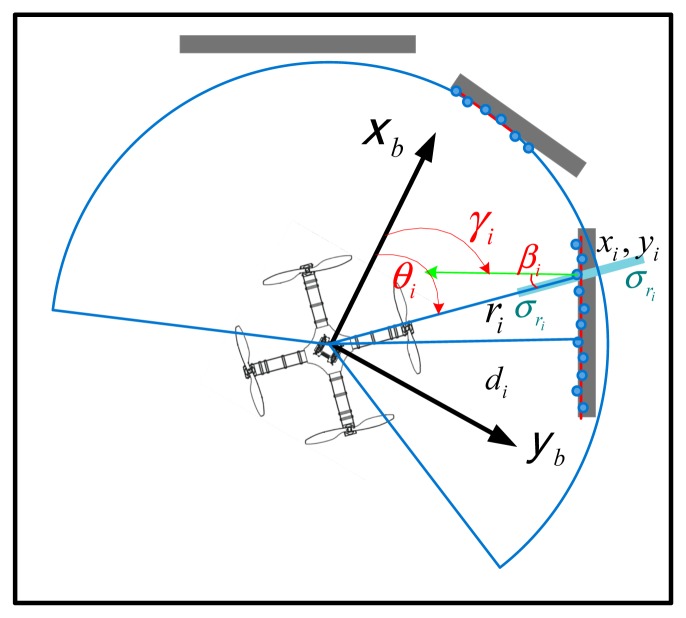
Uncertainty analysis of a laser range measurement. The blue points denote the measurement points in a single laser scan at time *t. γ* is the angle between the body axis and the vector normal of the surface at measurement point (*x_i_*, *y_i_*), and *β_i_* is the angle form the vector normal to the measurement direction. *d* is the distance from the origin to the line segment *i*.

For a quadrotor using a laser rangefinder, the information matrix *N* in Equation (11) denotes the information provided by laser scans, which can be calculated by combining the information of each range measurement of the environment. Considering an environment geometry shown in Figure 5, a laser scan obtained at time *t* consists of measurements of *n* scan points in the environment, each of which is described by a range reading *r_i_* and a measurement angle *θ_i_*, and the information provided in a laser scan (*r_i_*, *θ_i_*) is in the direction perpendicular to the topology, *i.e.*, the direction of the vector normal (the vector in green, Figure 5) of the environment surface. Therefore, the information matrix can be obtained by projecting the range information onto the vector normal of the surface and summing the projected information of each scan point. The information matrix *N* can be calculated by:

(44) $$\text{N}≜{\text{H}}^{T}{\left({\text{∑}}_{r}^{2}\right)}^{-1}\text{H}=\left[\begin{array}{ccc} {\text{∑}}_{xx} & {\text{∑}}_{xy} & {\text{∑}}_{x\psi }\\ {\text{∑}}_{xy} & {\text{∑}}_{yy} & {\text{∑}}_{y\psi }\\ {\text{∑}}_{x\psi } & {\text{∑}}_{y\psi } & {\text{∑}}_{\psi \psi } \end{array}\right]$$

with **H** as the measurement matrix (Equation (45)) and ${\text{∑}}_{r}^{2}$ as the variance matrix (Equation (46)) denoting the variance of each measurement:

(45) $$\text{H}=\left[\begin{array}{ccc} \cos\gamma_{1}\cos\left(\gamma_{1}-\theta_{1}\right) & \sin\gamma_{1}\cos\left(\gamma_{1}-\theta_{1}\right) & r_{1}\sin\left(\gamma_{1}-\theta_{1}\right)\\ \vdots & \ddots & \vdots \\ \cos\gamma_{n}\cos\left(\gamma_{n}-\theta_{n}\right) & \cos\gamma_{n}\cos\left(\gamma_{n}-\theta_{n}\right) & r_{n}\sin\left(\gamma_{n}-\theta_{n}\right) \end{array}\right]$$

(46) $${\text{∑}}_{r}^{2}=\text{diag}\left(\sigma_{r_{1}}^{2},\sigma_{r_{1}}^{2},\sigma_{r_{1}}^{2},\ldots \sigma_{r_{i}}^{2},\sigma_{ri}^{2},\sigma_{r_{i}}^{2}\ldots \sigma_{r_{n}}^{2},\sigma_{r_{n}}^{2},\sigma_{r_{n}}^{2}\right)$$

In our experiments, the readings of each measurement point (*r_i_*, *θ_i_*) are obtained first from the simulated laser scan, and then, the line segments of the topology surface are extracted using the measurement points, along with the vector normal of each line segments, as well as the angle between the body axis and the vector normal. From Equations (44)–(46), the information matrix *N* can be calculated with the summation terms that are given as follows:

$$\begin{array}{l} {\text{Σ}}_{xx}=\overset{m}{\underset{i}{\text{∑}}}\frac{\cos^{2}\gamma_{i}\cos^{2}(\gamma_{i}-\theta_{i})}{\sigma_{r_{i}}^{2}},{\text{Σ}}_{yy}=\overset{m}{\underset{i}{\text{∑}}}\frac{\sin^{2}\gamma_{i}\cos^{2}(\gamma_{i}-\theta_{i})}{\sigma_{r_{i}}^{2}}\\ {\text{Σ}}_{\psi \psi }=\overset{m}{\underset{i}{\text{∑}}}\frac{r_{i}^{2}\sin^{2}(\gamma_{i}-\theta_{i})}{\sigma_{r_{i}}^{2}},{\text{Σ}}_{xy}=\overset{m}{\underset{i}{\text{∑}}}\frac{\cos\gamma_{i}\sin\gamma_{i}\cos^{2}(\gamma_{i}-\theta_{i})}{\sigma_{r_{i}}^{2}}\\ {\text{Σ}}_{x\psi }=\overset{m}{\underset{i}{\text{∑}}}\frac{r_{i}\cos\gamma_{i}\sin(\gamma_{i}-\theta_{i})\cos(\gamma_{i}-\theta_{i})}{\sigma_{r_{i}}^{2}},{\text{Σ}}_{y\psi }=\overset{m}{\underset{i}{\text{∑}}}\frac{r_{i}\sin\gamma_{i}\sin(\gamma_{i}-\theta_{i})\cos(\gamma_{i}-\theta_{i})}{\sigma_{r_{i}}^{2}} \end{array}$$

The above analysis provides a statistical approach to incorporating the variance of the laser scan data into the uncertainty of the pose estimate. Therefore, we can use Equations (44)–(46) to calculate the information matrix *N*, which is used in the linear covariance propagation as described in Equation (11).

**Figure 6.**
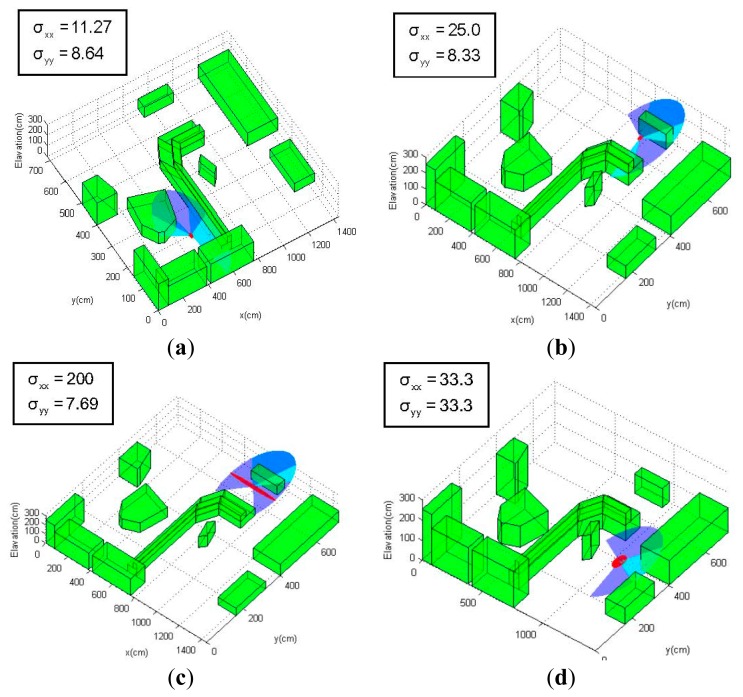

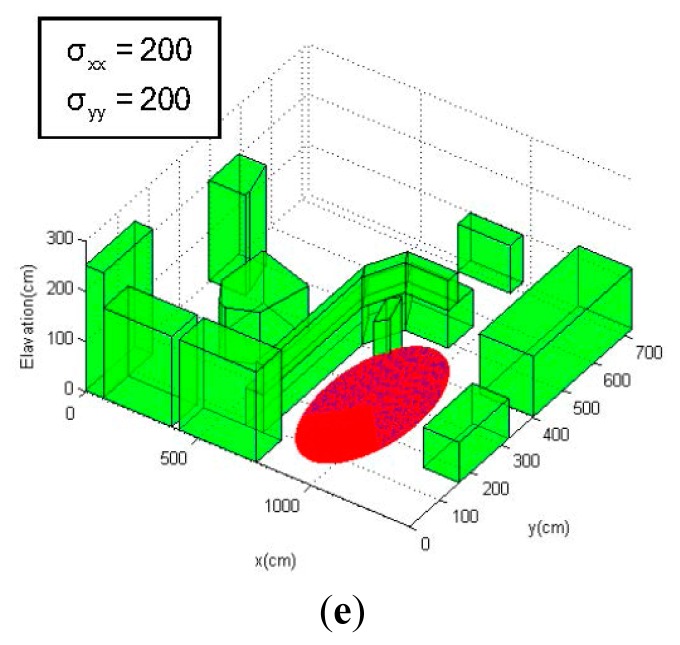
Position estimation uncertainties based on a simulated laser sensor along a predefined path. Cyan sectors denote the laser data with valid environmental measurements. Red ellipses illustrate the 1–*σ* localization covariance; larger ellipses indicate high uncertainty poses. All covariance are in cm. (**a**) position: (5.0, 2.0, 0.5) m, heading: −45°; (**b**) position: (8.0, 7.5, 0.5) m, heading: −45°; (**c**) position: (8.0, 7.5, 0.5) m, heading: 0°; (**d**) position: (11.0, 4.0, 1.0) m, heading: −30°; (**e**) position: (10.0, 2.5, 1.0) m, heading: 0°.

Figure 6a–e show the uncertainty ellipses related to the position estimation uncertainties along a predefined path calculated using the information matrix equations proposed in this section, as well as the covariance propagation approaches in Section 4. In the experiments, an 18 × 9 m 3D environment model is derived from a cluttered real-world laboratory, and the MAV is assumed to be equipped with a simulated laser rangefinder that is able to generate range and bearing measurements of the environment. In order to highlight the influences of the environment, the laser sensor model is assumed to have a limited sensing capability with a 2-m range and a 240° field-of-view, which is represented by blue sectors in the figures (Although this sensor model is unrealistic in terms of maximum range, it serves well to illustrate how the environment and sensor measurement affect the localization uncertainty). As the MAV navigates along the path and rotates its orientations, the environment topologies that fall within the field-of-view will generate a series of noisy measurements of the topologies' distance and bearing to the MAV body (the valid measurements are demonstrated by the cyan sectors in the following figures), enabling the MAV to localize itself by a EKF-based approach using these measurements of the environment. The 1–σ position uncertainty is demonstrated by red ellipses in the figures, and the size and shape of the ellipses indicate the relative scale of the localization uncertainty in both *x* and *y* directions.

As can be seen from Figure 6, the area where the onboard sensor is expected to detect more environmental topologies tends to produce position estimates with low uncertainty (Figure 6a,b), while those locations where the sensor only encounters just one obstacle will lead to high localization uncertainty (Figure 6d). In extreme cases where the MAV navigates into wide open areas (Figure 6e), the localization uncertainty increases sharply, since these areas provide almost no information for localization. In addition, the localization uncertainty is also affected by the orientation (yaw) of the MAV relative to the environmental topologies: The MAV is at the same *x*-*y*-*z* coordinates in Figure 6b,c, but the position uncertainty varies due to the different yaw orientations of the sensor (−45° in Figure 6b and 0° in Figure 6c); the yaw angle is positive when the rotation is counterclockwise around the *z*-axis of the Earth-fixed frame). The environmental topology of the MAV location in Figure 6b,c can be considered as a corridor between two parallel obstacles, and the laser sensor measurements will provide most information (large information matrix *N*) in the direction perpendicular to the local environment, according to the model given in Equations (44)–(46). As a result, the uncertainty in the direction of the “corridor” (*x* direction of the environment frame) is much larger when the measurement direction is perpendicular to the “corridor” (Figure 6c).

### 6.5. Numerical Simulation Results

#### 6.5.1. Scenario 1: Indoor Environment with Open Space

**Figure 7.**
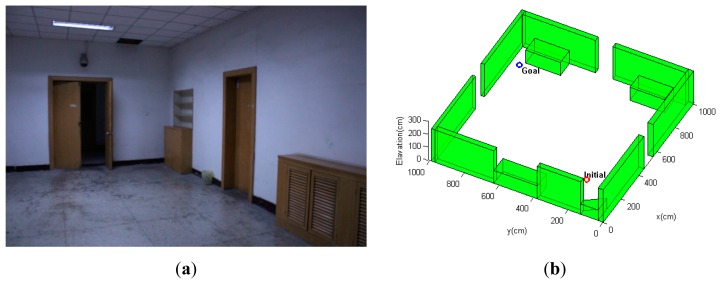
Indoor environment simulation Scenario 1: (**a**) A hallway of Tsinghua University's main building; (**b**) simulated 3D model of the GPS-denied indoor environment. The red and blue circles indicate the initial and goal location of the MAV, respectively.

The environment of the first scenario is shown in Figure 7a. As can be seen from Figure 7, this scenario is a 10 × 10-m open indoor environment with all structures concentrated along the peripheral walls, and the center of the environment is an open region that is out of the measurement range of the simulated laser rangefinder. The MAV quadrotor must navigate from the initial position at the bottom-right corner through the hallway to a goal region, which is diagonally opposite of the initial point. Since most of the environment provides rare measurement for localization, it is even more critical for the path planning strategy to find paths that maximize information gain and reduce state uncertainty; hence, the emphasis of this simulation scenario is to test the path planning algorithm's ability to generate paths that ensure the MAV's localization performance throughout the path. For comparison purposes, the conventional CL-RRT algorithm without the incorporation of state uncertainty is also tested using the same MAV model and environment configurations.

In this scenario, the quadrotor MAV begins at **x***_initial_* =[2.0 2.0 0.0]*^T^* m. It is intended to navigate towards the goal location at **x***_goal_* =[9.0 9.0 1.0]*^T^*m, and the weight factors in Equation (19) are set as: *ζ*_1_ = 1, *ζ*_1_ = 1, *ζ*_3_ = 100. The cascaded PID control scheme described in Section 6.2 is applied for both CL-RBT state propagation and reference path following of the MAV. The planning-execution cycle is set to 5 s.

**Figure 8.**
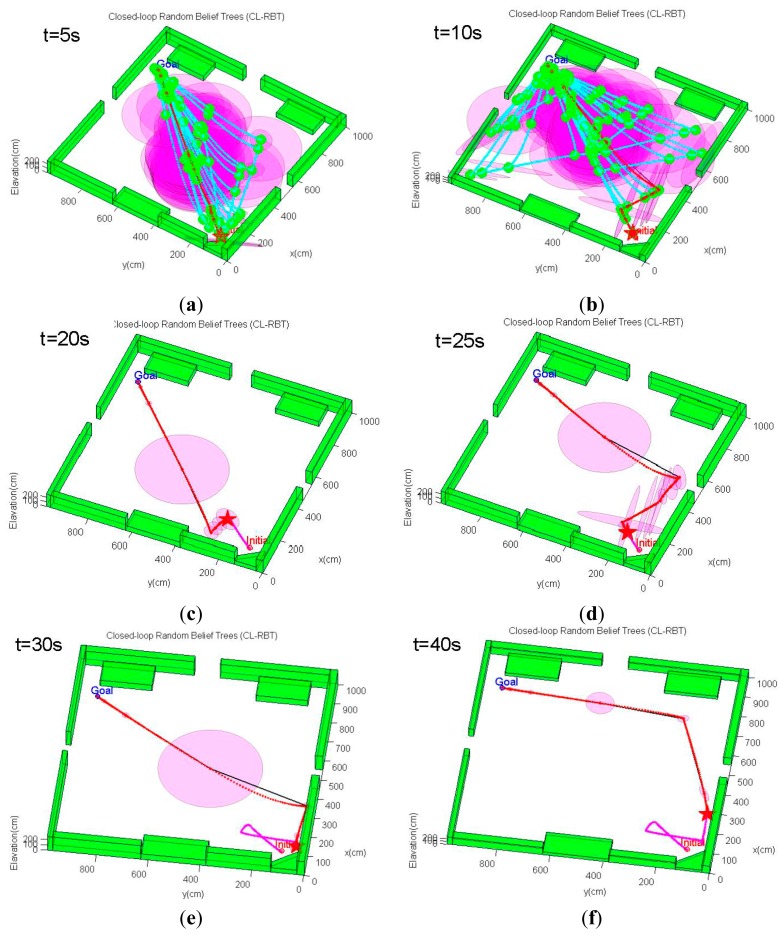

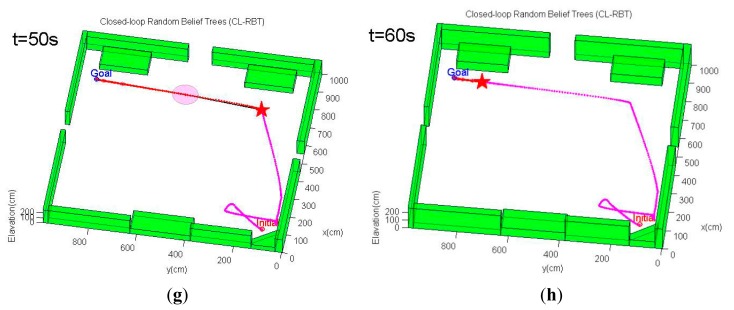
Example results of trajectory generated by CL-RBT for a quadrotor MAV navigating in simulation Scenario 1. The pink ellipses represent the covariance of the position estimate, and large ellipses denote MAV states with high uncertainty. The current state (MAV's 3D positions and headings) is denoted with a red pentacle. The current selected path (specified by node sequence, used as the reference to the control system) is marked in black with red dots representing the nodes. The predicted closed-loop trajectory corresponding to the current selected node sequence is emphasized in red, while the actual output trajectory flown by the closed-loop MAV model is marked in pink. The current tree is denoted by green nodes and cyan trajectories, but is set as invisible after (**b**) for clarity. (**a**) t = 5 s; (**b**) t = 10 s; (**c**) t = 20 s; (**d**) t = 25 s; (**e**) t = 30 s; (**f**) t = 40 s; (**g**) t = 50 s; (**h**) t = 60 s.

Figure 8 demonstrates an example trajectory generated by a trial of the simulation scenario. The CL-RBT algorithm quickly generates a feasible path that goes through the regions with as much measurements as possible to localize and reaches the goal (Figure 8a,b). Since a comparatively small amount of samples and nodes are generated during the initial planning, the initially selected paths may still reach the open regions with comparatively high localization uncertainty (Figure 8c–e). However, as more samples and nodes are added, the CL-RBT continuously refines the path in real time, identifying optimal paths that go through measurement-rich regions to ensure localization and reduce the execution cost of the goal (Figure 8f–h).

**Figure 9.**
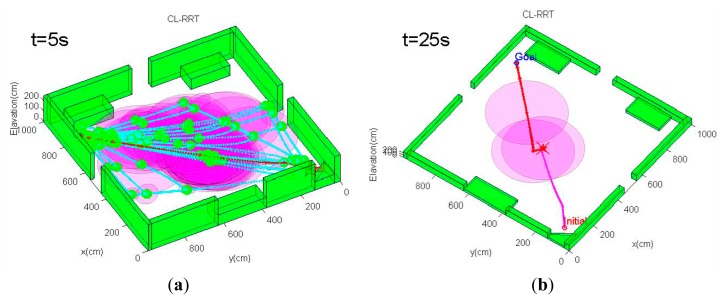
Example results of the trajectory generated by the conventional closed-loop rapidly exploring random trees (CL-RRT) for a quadrotor MAV navigating in simulation Scenario 1. The red cross in (**b**) denotes the state where the MAV's state estimation fails, since the position uncertainty has become sufficiently high. (**a**) t = 5 s; (**b**) t = 25 s.

In contrast, by expanding the path using the conventional CL-RRT and checking obstacle collision constraints, the resulting paths will move the MAV straight to the goal (Figure 9). Although the conventional CL-RRT may generate shorter paths, ignoring the localization factors will result in high localization uncertainty, since these paths may go through the open regions. In this case, following these paths would likely cause operation failure, since the state estimate becomes unacceptably uncertain, such that the MAV control would have become unstable.

#### 6.5.2. Scenario 2: Cluttered 3D Indoor Environment

**Figure 10.**
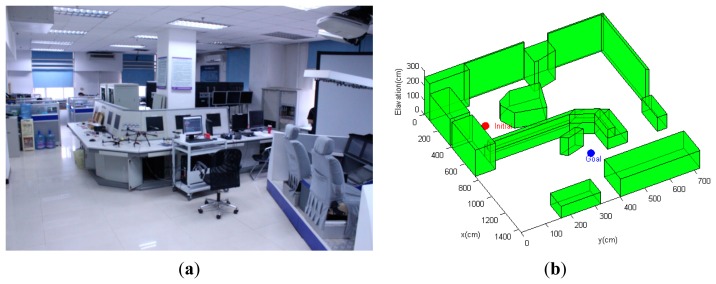
Indoor environment simulation Scenario 2: (**a**) view of a typical cluttered laboratory; (**b**) simulated 3D model of the unstructured, GPS-denied indoor environment. The red and blue dot indicate the initial and goal location of the MAV, respectively.

This scenario considers a more complex three-dimensional, GPS-denied indoor environment with a number of non-convex obstacles and structures (Figure 10). The environment model is derived from an actual cluttered laboratory, which is the same as the model shown in Figure 6. The objective of the quadrotor MAV is to navigate from the bottom-left initial point to the goal position located on the other side of the laboratory, moving safely between the obstacles. Planning trajectories in this cluttered environment is a challenging task for the path planner. The unstructured environment requires the path planning algorithm to be able to generate a trajectory that avoids the non-convex obstacles to ensure feasibility, while bounding the state estimate uncertainty along the trajectory to allow the MAV to remain well-localized.

For this scenario, the quadrotor MAV begins from one side of the environment at **x***_initial_* =[3.2 6.4 0.0]*^T^*, and it is intended to navigate to the goal point behind a cluttered region of obstacles at **x***_goal_* =[10.0 4.0 0.5]*^T^m*. The same MAV dynamics model, PID control scheme and sensor configuration as in Scenario 1 are also applied in this scenario. However, we select weighting factors in Equation (19) as *ζ*_1_ = 1, *ζ*_1_ = 20, *ζ*_3_ = 30, since the emphasis of this scenario is on generating feasible trajectories that quickly reach the goal while reducing localization uncertainty. The planning cycle in this scenario is extended to 7 s. The conventional CL-RRT is also tested using the same scenario and MAV system model for comparison purpose.

**Figure 11.**
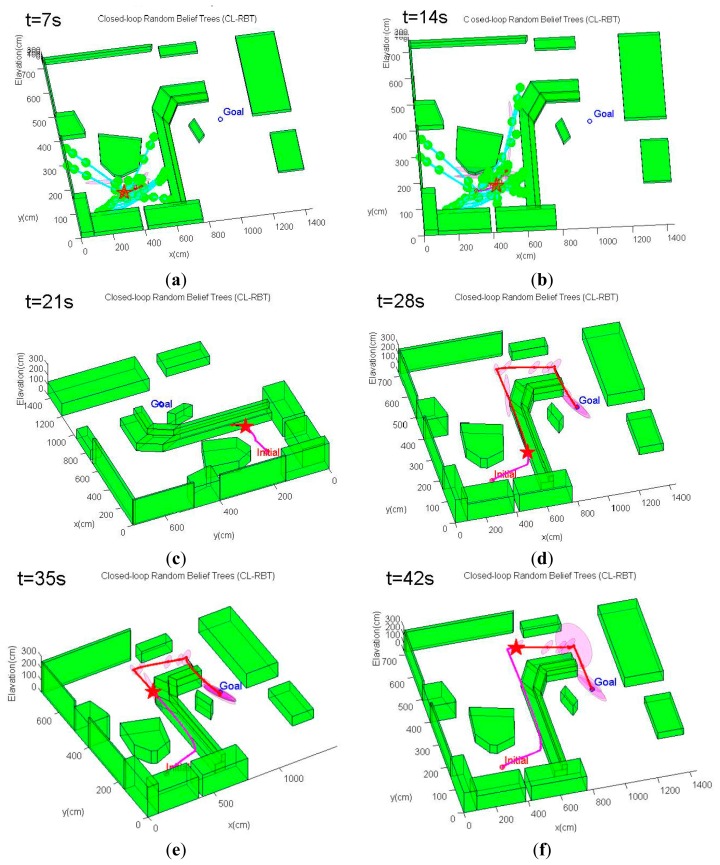

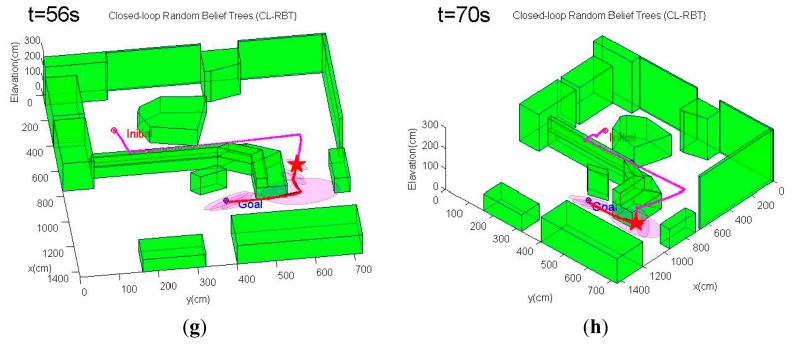
Example results of the trajectory generated by the CL-RBT for a quadrotor MAV navigating in a cluttered environment. The full legend for the symbols can be found in Figure 8. The current tree is set as invisible after (**b**) for clarity. (**a**) t = 7 s; (**b**) t = 14 s; (**c**) t = 21 s; (**d**) t = 28 s; (**e**) t = 35 s; (**f**) t = 42 s; (**g**) t = 56 s; (**h**) t = 70 s.

An example of the trajectory simulation results from this scenario is depicted in Figure 11. As can be seen from Figure 11, the MAV's initial position is located in an obstacle-rich region. The path planner initially has to move the MAV out of this cluttered region. Unlike the first scenario, the CL-RBT algorithm does not find any trajectories directly to the goal due to the poor coverage of the CL-RBT tree and the cluttered environment in the first few planning cycles (Figure 11a,b). As the CL-RBT tree expands to cover more space after some wandering, the CL-RBT algorithm moves the MAV progressively through the narrow passages and approaches towards the direction of the goal region Figure 11c). After moving out of the narrow passage region, the path planner succeeds in identifying a trajectory to the goal (Figure 11d). As can be seen from the figure, the covariance of the position estimate stays bounded along the trajectory during the entire planning cycles of CL-RBT.

**Figure 12.**
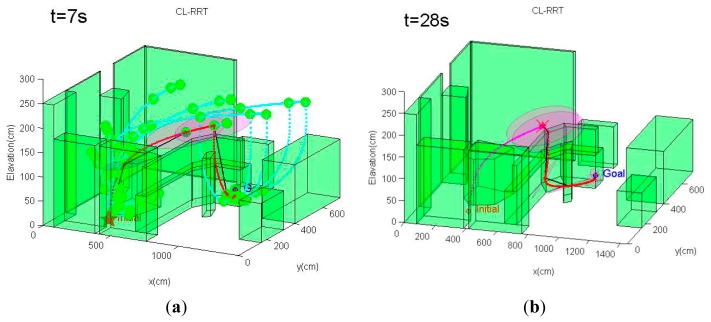
Example results of trajectory generated by the conventional CL-RRT for a quadrotor MAV navigating in simulation Scenario 1. The red cross in (**b**) denotes the position where the MAV's state estimation fails since the position uncertainty have exceeded the threshold. (**a**) t = 7 s; (**b**) t = 28 s.

In contrast to CL-RBT, the conventional CL-RRT quickly finds a path that goes over the cluttered obstacle region and directly reaches the goal (Figure 12a). Although the resulting path is shorter than the path generated by CL-RBT, the uncertainty of the position estimate grows rapidly along the CL-RRT trajectory (Figure 12b); this is because the environment becomes open as the MAV's altitude increases, providing rare information to the onboard sensor for localization.

#### 6.5.3. Performance Comparison and Analysis

In order to illustrate the performance of the CL-RBT algorithm, we compare the performance statistics of the CL-RBT algorithm to that of the conventional CL-RRT by running simulation experiments using both algorithms on the same scenarios, as described in Sections 6.5.1 and 6.5.2 (note that we did not compare the CL-RBT to other conventional sampling-based path planners that operate on static graphs or trees, such as PRM, RRT or BRM, since they are not real-time algorithms in essence). For each simulation scenario, the experiment is repeated 30 times using each of the two algorithms, and the performance statistics is recorded and evaluated in terms of the paths'; cost (total length) and the trace of the MAV's expected covariance at the goal when following the generated paths, both averaged over 30 times.

Table 1 shows a comparison of the average path length and covariance statistics of CL-RBT and conventional CL-RRT. The CL-RBT outperforms conventional CL-RRT in terms of expected uncertainty at the goal in both simulation scenarios. Moreover, since these experiments are performed in simulation, the actual trajectories of the MAV are available, and the average mean error between the estimated MAV position and the actual position at the goal location are also computed and shown in Table 1 (note that the mean errors corresponding to the conventional CL-RRT are computed by assuming that the controller is able to execute the prescribed path and navigate the MAV to the goal location regardless of the estimation error, while in fact, the MAV controller would have failed halfway). These results indicate that the conventional CL-RRT results in large deviations from the true position, while the CL-RBT can effectively bound the estimation error in repeated trials. However, the resulting paths generated by CL-RRT are generally longer that those of conventional CL-RRT. This is because CL-RBT integrates the prediction of position uncertainty into the planning progress, which enabling the MAV to balance minimizing path length against reducing localization uncertainty, selecting longer paths to avoid regions that lead to high position estimation uncertainty when necessary.

**Figure 13.**
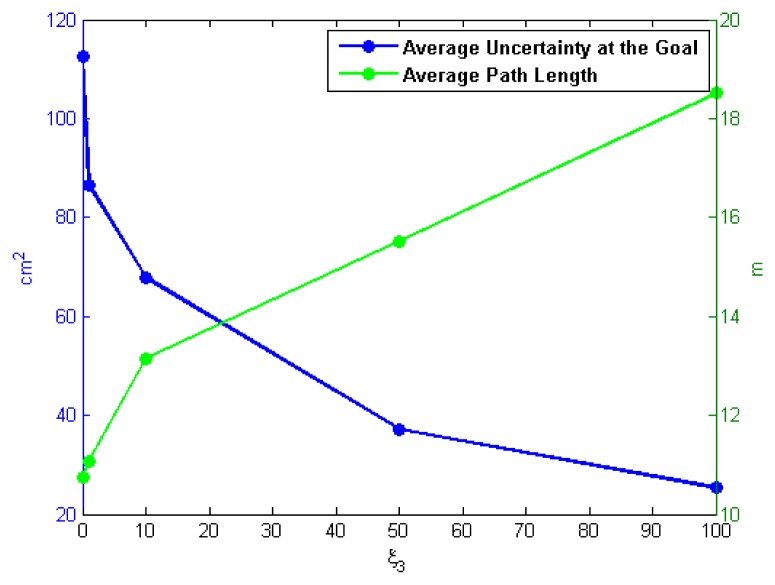
Comparison of average path length and average uncertainty cost at the goal. Data points correspond to different values of the uncertainty cost weighting factor (Equation (19)): *ζ*_3_ = {0, 1, 10, 50, 100}.

As discussed previously, the specification of weighting factors in the cost function for path selection Equation (19) is an important factor for implementations of CL-RBT. To evaluate the effect of the selection on weighting factors, we have conducted a number of simulations with different values of weighting factor *ζ*_3_ in Scenario 1. Five different values of *ζ*_3_ are evaluated in these simulations: *ζ*_3_ = {0, 1, 10, 50, 100}. For each of the five values, the simulation is repeated 10 times, and the path length and uncertainty cost (trace of the covariance) at the goal are recorded and averaged. Figure 13 illustrates the resulting paths' average length and uncertainty cost at the goal as a function of *ζ*_3_. As can be seen from the figure, the uncertainty cost decreases as the weighting factor *ζ*_3_ increases, whereas the path becomes comparatively longer. This is because reducing localization uncertainty becomes relatively more significant as *ζ*_3_ increases, resulting in longer paths that take more measurements from the environment. These results demonstrate that the weighting factors of the cost function should be carefully selected depending on the specific task requirement and the characteristic of the environment.

## 7. Conclusions and Future Work

This paper presents a real-time path planning approach in belief space (CL-RBT) for MAVs with complex dynamic constraints under state estimation uncertainty. The proposed CL-RBT approach is built upon the RRT motion planning framework. We made two primary contributions in this paper. First, the prediction of state uncertainty is incorporated in the path planning process, which allows the path planning strategy to find the path that reduces the state uncertainty and ensures state estimation confidence, using an efficient, linear update process of covariance in a factored form. Second, closed-loop prediction is used in the motion planning framework to generate dynamically feasible trajectories, enabling the motion planner to handle complex dynamic and environment constraints. Simulation results demonstrate that the motion planner can be implemented in real time, generating dynamically feasible paths that trade off minimizing path cost and reducing state uncertainty accuracy, while easily handling complex vehicle dynamics. It can also be concluded that CL-RBT has the potential to increase the autonomy of MAVs that operate in complex, GPS-denied environments.

While the utility and advances of the CL-RBT algorithm have been presented in this paper, there still remains significant future work on the proposed framework. Future work will focus on the more extensive analysis of the algorithm's performance, including the computational complexity and optimality of the generated paths. Further theoretical work is also necessary in the proof and analysis of the algorithm's completeness and convergence. Moreover, we are also planning to implement the CL-RBT on an actual quadrotor MAV platform that is currently under development. It would be possible to validate the performance of CL-RBT with realistic onboard sensors (laser rangefinders, RGB-D, cameras, *etc.*) through flight tests in actual indoor environments.

## Figures and Tables

**Table 1.** Performance comparisons of CL-RBT and CL-RRT, using 30 simulation runs.

**Algorithms**	**Parameters**	**Scenario 1**	**Scenario 2**
**CL-RBT**	Path length (m)	17.35	16.7
	Final covariance (cm^2^)	**39.88**	**41.65**
	Final mean error (m)	1.07	0.82

**CL-RRT**	Path length (m)	10.74	12.78
	Final covariance (cm^2^)	5,933	9,523
	Final mean error (m)	54.33	73.52

## References

[1] Bachrach A., He R., Roy N. (2009). Autonomous Flight in Unknown Indoor Environments. Int. J. Micro Air Veh..

[2] Shaojie S., Michael N., Kumar V. (2012). Autonomous Indoor 3D Exploration with a Micro-Aerial Vehicle.

[3] Bry A., Bachrach A., Roy N. (2012). State Estimation for Aggressive Flight in GPS-denied Environments Using Onboard Sensing.

[4] Abdelkrim N., Aouf N., Tsourdos A., White B. (2008). Robust Nonlinear Filtering for INS/GPS UAV Localization.

[5] Michael N., Fink J., Kumar V. (2010). Cooperative Manipulation and Transportation with Aerial Robots. Auton. Robot..

[6] Chowdhary G., Sobers D.M., Pravitra C., Christmann C., Wu A., Hashimoto H., Ong C., Kalghatgi R., Johnson E.N. (2012). Self-Contained Autonomous Indoor Flight with Ranging Sensor Navigation. J. Guid. Control Dyn..

[7] Weiss S., Scaramuzza D., Siegwart R. (2011). Monocular-SLAM based Navigation for Autonomous Micro Helicopters in GPS-denied Environments. J. Field Robot..

[8] Huang A.S., Bachrach A, Henry P., Krainin M., Maturana D., Fox D., Roy N. Visual Odometry and Mapping for Autonomous Flight Using an Rgb-d Camera.

[9] Frazzoli E., Dahleh M.A., Feron E. (2002). Real-time motion planning for agile autonomous vehicles. J. Guid. Control Dyn..

[10] Samuel P., Roy N. (2009). The Belief Roadmap: Efficient Planning in Belief Space by Factoring the Covariance. Int. J. Robot. Res..

[11] Thrun S., Burgard W., Fox D. (2005). Partially Observable Markov Decision Process. Probabilistic Robotics.

[12] Kaelbling L., Littman M., Cassandra A. (1998). Planning and Acting in Partially Observable Stochastic Domains. Artif. Intell..

[13] Berg V.D., Patil J.S., Alterovitz R. (2012). Motion Planning under Uncertainty Using Iterative Local Optimization in Belief Space. Int. J. Robot. Res..

[14] Bai H., Hsu D., Lee W., Ngo V. (2011). Monte Carlo Value Iteration for Continuous State POMDPs.

[15] Kavraki L.E., Svestka P., Latombe J.C., Overmars M.H. (1996). Probabilistic Roadmaps for Path Planning in High-Dimensional Configuration Spaces. IEEE Trans. Robot. Autom..

[16] LaValle S.M. (1998). Rapidly-Exploring Random Trees: A New Tool for Path Planning.

[17] Kuwata Y., Teo J., Karaman S., Fiore G., Frazzoli E., How J.P. Motion Planning in Complex Environments Using Closed-loop Prediction.

[18] Kuwata Y., Teo J., Fiore G., Karaman S., Frazzoli E., How J.P. (2009). Real-time motion planning with applications to autonomous urban driving. IEEE Trans. Control Syst. Technol..

[19] Berg V.D., Abbeel P., Goldberg K. (2011). LQG-MP: Optimized Path Planning for Robots with Motion Uncertainty and Imperfect State Information. Int. J. Robot. Res..

[20] Bry A., Roy N. (2011). Rapidly-exploring Random Belief Trees for Motion Planning under Uncertainty.

[21] He R., Prentice S., Roy N. (2008). Planning in Information Space for a Quadrotor Helicopter in a Gps-denied Environment.

[22] Abraham B., Prentice S., He R., Henry P., Huang A.S., Krainin M., Maturana D., Fox D., Roy N. (2012). Estimation, Planning, and Mapping for Autonomous Flight Using an RGB-D Camera in GPS-denied Environments. Int. J. Robot. Res..

[23] Levine D., Luders B., How J.P. Information-rich path planning with general constraints using rapidly-exploring random trees.

[24] Kalman E.R. (1960). A New Approach to Linear Filtering and Prediction Problems. J. Fluid. Eng..

[25] Smith R., Self M., Cheeseman P., Cox I.J., Wilfong G.T. (1990). Estimating Uncertain Spatial Relationships in Robotics. Autonomous Robotic Vehicles.

[26] Qing L., Dachuan L., Qifan W., Liangwen T., Yan H., Yixuan Z., Nong C. (2013). Autonomous navigation and environment modeling for MAVs in 3-D enclosed industrial environments. Comput. Ind..

[27] Park S., Deyst J., How J.P. (2007). Performance and Lyapunov Stability of a Nonlinear Path-Following Guidance Method. J. Guid. Control Dyn..

[28] Bachrach A., Prentice S., He R., Roy N. (2011). RANGE—Robust autonomous navigation in GPS-denied environments. J. Field Robot..

[29] Bachrach A. (2009). Autonomous Flight in Unstructured and Unknown Indoor Environments. Master's Thesis.

